# Influence of alkali metals on water dynamics inside imidazolium-based ionic liquid nano-domains

**DOI:** 10.3389/fchem.2022.1028912

**Published:** 2022-11-15

**Authors:** Katarzyna Dziubinska-Kühn, Mina Maddah, Marion Pupier, Jörg Matysik, Jasmine Viger-Gravel, Magdalena Kowalska, Beatrice Karg

**Affiliations:** ^1^ CERN, Geneva, Switzerland; ^2^ Institute of Analytical Chemistry, University of Leipzig, Leipzig, Germany; ^3^ Department of Chemistry, K.N.Toosi University of Technology, Tehran, Iran; ^4^ Department of Organic Chemistry, University of Geneva, Geneva, Switzerland; ^5^ Department of Nuclear and Particle Physics, University of Geneva, Geneva, Switzerland

**Keywords:** ionic liquid, structural arrangement, water, NMR, molecular dynamics

## Abstract

The global need to expand the design of energy-storage devices led to the investigation of alkali metal - Ionic Liquid (IL) mixtures as a possible class of electrolytes. In this study, 1D and 2D Nuclear Magnetic Resonance (NMR) and Electrochemical Impedance Spectroscopy (EIS) as well as Molecular Dynamics (MD) simulations were used to study the intermolecular interactions in imidazolium-based IL - water - alkali halide ternary mixtures. The ^1^H and ^23^Na 1D and ^1^H DOSY NMR spectra revealed that the presence of small quantities of NaCl does not influence the aggregation of water molecules in the IL nano-domains. The order of adding ionic compounds to water, as well as the certain water and NaCl molecular ratios, lead to the formation of isolated water clusters. Two ternary solutions representing different orders of compounds mixing (H_2_O+ IL + NaCl or H_2_O+ NaCl + IL) showed a strong dependence of the initial solvation shell of Na^+^ and the self-clustering of water. Furthermore, the behaviour of water was found to be independent from the conditions applied during the solution preparation, such as temperature and/or duration of stirring and aging. These findings could be confirmed by large differences in the amount of ionic species, observed in the ternary solutions and depending on the order of mixing/solute preparation.

## 1 Introduction

Advancing energy-storage technologies inherently face the challenge of limited lithium resources. Hence, the development of new classes of rechargeable alkali-ion batteries becomes a viable long-term trend in electrochemistry and material science. Among all the alkali metals, the most abundant are sodium and potassium, the new likely candidates to replace lithium in alkali metal-ion batteries [Abraham (2020); Sun et al. (2019; 2020); Xu et al. (2020)]. Additionally, new types of non-aqueous electrolytes, based on ionic liquids (ILs), are being investigated to not only increase the feasibility of alkali metal-based energy-storage devices, but also to reduce their environmental impact [Tong et al. (2020); McEldrew et al. (2021a); Hwang et al. (2021)].

Because the storage capacity of a battery results directly from the number and mobility of the ions present in the electrolyte, ILs gained recent attention, due to their purely ionic structure [Marczewski et al. (2014); Hakim et al. (2021)]. Moreover, selection of ILs can result in low vapour pressure of the solution, and consequently, reduced or no flammability [Bellusci et al. (2020)]. A popular family of solvents are imidazolium (C_*n*_MIM)-based ionic liquid, where two protons in the aromatic ring of the cation are substituted with a methyl group and alkyl chain [Tong et al. (2020); Seki et al. (2007)].

The properties of the ILs are significantly influenced by the length of the side alkyl chain (tail). This is a direct result of the significant differences in their molecular arrangement. For instance, the overall liquid kinetics are dominated by hydrophobic interactions between the long (hydrophobic) alkyl chains, if the number of carbon atoms n_*C*_ ≥ 6 [Canongia Lopes and Pádua (2006)]. This formation of micelle-like non-polar nano-domains is independent of the IL anion, and can be confirmed using x-ray diffraction (XRD) or small-angle scattering (SAXS) spectra [Bruce et al. (2017); Triolo et al. (2007); Shimizu et al. (2013)].

Reducing the number of carbon atoms in the side chains results in weaker tail-tail interactions, and thus, promotion of the *π* - *π* stacking and cation-anion Coulomb interactions [Singh and Kumar (2007); Katayanagi et al. (2004)]. Consequently, the final IL network is based on the formation of polar and not non-polar (as for n_*C*_ ≥ 6) nano-domains, predominantly influenced by the selection of small and hydrophilic anions without steric hindrance [Yoshida et al. (2007)]. Hence, selected short-side alkyl chains ILs, or functionalized long-side alkyl chains without hydrophilic tails, can form recurring polar nano-domains within the solvent network [Triolo et al. (2012); Canongia Lopes and Pádua (2006)]. The formation of non-polar nano-domains based on tail-to-tail aggregation is not observed, in contrast to widely confirmed micelle formation for C_*n*_MIMs with n_*C*_ ≥ 6 [Vazquez-Salazar et al. (2020); Russina et al. (2009)]. Importantly, the dispersion of the short tails has no longer random character, if the IL is doped with water. Recent studies revealed the presence of distinguished small water clusters, repetitively surrounded by polar and non-polar IL environment if n_*C*_ = 2 [Saihara et al. (2015); Dziubinska-Kühn et al. (2021)], or n_*C*_ = 4 [Gao and Wagner (2016)]. Moreover, the rigidness of the IL cation-anion arrangement leads to the induced distortion of small water aggregates, instead of their self-aggregation into larger structures [Kashin et al. (2016); Ivanov et al. (2021)].

Designing a new imidazolium-based electrolyte is frequently based on the direct mixing of the IL and a molecular fraction of alkali or neutral inorganic salt, based on the metal of choice, both containing the same anion [Becker et al. (2021); Gouverneur et al. (2018)]. During further properties evaluation, this selection allows to distinguish between the cation’s and anion’s role in the ion mobility [Aguilera et al. (2015)]. However, the mutual anion oversaturation might lead to exclusive metal (M) cation solvation, resulting in the opposite charge of the M^+^-based species, which decreases the overall conductivity of the electrolyte [McEldrew et al. (2021b); Molinari et al. (2019)]. Thus, to maintain good electrochemical properties of the solution, avoiding the excess presence of a bulky IL anion should be considered. A possible way to achieve it, is to select small-size metal salts, such as halides (X) [Gupta et al. (2019); Sieffert and Wipff (2007)]. Due to their size, they will not disturb the characteristic molecular arrangement of imidazolium-based ILs with the short side alkyl chain, but will be incorporated in the heterogenic polar nano-domains [Kuzmina et al. (2016); Pereiro et al. (2012b); Aguilera et al. (2015)].

The battery feasibility can be characterised using ion mobility, transference number and general transfer properties, directly resulting from the structural arrangement and solvation patterns observed in the electrolyte [McEldrew et al. (2021a); Zhang and Maginn (2015); Abraham (2020)]. Adding an inorganic salt to IL can increase the charge density of the electrolyte, and thus, its ionicity, while preserving the unique character of ionic liquid being the primary solvent [Pereiro et al. (2012b,a)]. Nevertheless, the highly organized IL network can enhance formation of neutral ion pairs and/or large aggregates, which do not participate in the overall conductivity of the electrolyte [Zhang and Maginn (2015); Kunze et al. (2011); Egashira et al. (2009)]. In addition, the presence of alkali-metal salts negatively affects the ion mobility, due to higher density and viscosity [Gouverneur et al. (2018); Lewandowski and Swiderska-Mocek (2009); Tong et al. (2020)]. A possible method to increase the number of ionic species in IL/inorganic salt mixtures is the addition of H_2_O. Because water molecules do not serve as charge carriers [Widodo et al. (2018)], the main advantage of their presence is dilution of the IL/inorganic salt aggregates, helping to reduce the mutual neutralization of ions [Widodo et al. (2018)].

A simultaneous incorporation of water and inorganic salt in the IL network allows to reduce the number of H_2_O molecules required to increase the overall conductivity [Becker et al. (2021); Miyazaki et al. (2019)]. As a consequence, it becomes easier to maintain the structural arrangement of the doped ionic liquid, and thus, preserve the properties of the primary solvent, while increasing the mobility of the secondary ionic species [Chaban (2015)].

**FIGURE 1:**
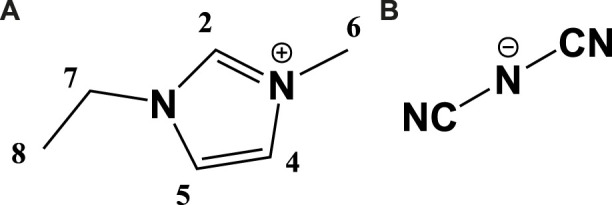
The structure and labeling pattern of 1-ethyl-3-methylimidazolium (EMIM) cation **(A)** and dicyanamide (DCA) anion **(B)**.

Herein, 1-ethyl-3-methylimidazolium dicyanamide (EMIM-DCA, Figure 1) - water - NaCl ternary system is selected as an example demonstrating the influence of IL - H_2_O self-structurization on final properties of a ternary solution. Because the behaviour of inorganic salts in the imidazolium-based ILs additionally depends on the properties of metal cation and inorganic anion (Amado-Gonzalez et al. (2017); Ghareh Bagh et al. (2013), each ternary solution should be carefully evaluated. Moreover, the presented analysis has a fundamental character, focusing on the importance of molecular arrangement in the theoretical electrolyte design. Towards the applications, this study should be followed by the analysis of the electrolyte’s charge/discharge cycling performance. The investigation of suitability of ternary solutions for energy-storage applications should also include the physicochemical properties of solid electrodes, frequently attenuated during the cycling life, e.g., due to the electrolyte degradation.

Although the advanced EMIM-DCA - water mutual relationship has been studied in detail (Dziubinska-Kühn et al. (2021), the influence of NaCl on the water and IL molecules inside polar and non-polar EMIM-DCA nano-domains is not yet understood. Hence, extensive analysis, based on varying IL:water:NaCl molecular ratio, is performed using nuclear magnetic resonance (NMR) spectroscopy to gain information about the structure and diffusivity of sodium cation within different types of solvation shells formed by EMIM-DCA and/or water. The occurrence and molecular ratio-dependence of ionic species, leading to enhanced electrochemical properties of the liquid, are discussed based on the overall conductance of the ternary solutions, determined with electrochemical impedance spectroscopy (EIS).

## 2 Experimental

### 2.1 Materials

Sodium chloride (purity > 99%), 1-ethyl-3-methylimidazolium dicyanamide (EMIM-DCA) (≥98.5%) and D_2_O (99.9%) were purchased from Sigma-Aldrich. H_2_O was deionized using a commercial Milli-Q purification system from Merck. Before the solution preparation, EMIM-DCA was dried in high vacuum environment (5.8 × 10^–5^ mbar, reached after 1 h) for 48–72 h. The water impurity was estimated below *X*_*w*_ ≤ 0.049 based on ^1^H NMR.

All samples were prepared at room temperature (RT) in atmospheric pressure condition, and measured within 24 h after preparation. Selected measurements were repeated within 1 h from the sample preparation, and using 6, 12, 24, and 48 h time increments, to confirm the kinetic stability of the observed results. The solutions were prepared according to the IL:water:NaCl molecular ratios presented in Supplementary Tables S1–S4. A vortex stirrer was used for selected time periods of 3–5 and 10 min to obtain homogeneous solutions. Due to the nature of the study, details of the compound mixing are specified at the beginning of each results and discussion subsection. For the summary see Table 1. In addition, two samples: neat EMIM-DCA and 0.1 M NaCl solution in water - EMIM-DCA mixture (*X*_*w*_ = 0.45) were exposed to the extensive time periods (from 5 to 60 min), where the surface to bulk liquid ratio was maximized for the used sample volume. Afterwards, ^1^H 1D NMR spectra were acquired to estimate the quantity of water absorbed by the sample as the function of time.

**TABLE 1:** Details of the mixing protocols applied during the sample preparation, specifying the mixing order (water + NaCl + IL and water + IL + NaCl), molar fraction of water (*X*_*w*_), mixing time, intermediate heating of the sample (from 298 K to 318 K) and temperature of the ^1^H 1D NMR measurements.

water + NaCl + IL			
*X*_*w*_	mixing time	intermediate heating	^1^H 1D NMR temperature
0.22	3–5 min	no	298 K
	3–5 min	yes	298 K, 318 K
	10 min	yes	298 K, 318 K
0.66	3–5 min	no	298 K
	3–5 min	yes	298 K, 318 K
	10 min	yes	298 K, 318 K
0.86	3–5 min	no	298 K
	3–5 min	yes	298 K, 318 K
	10 min	yes	298 K, 318 K
** water + IL + NaCl**			
0.22	3–5 min	no	298 K
	3–5 min	yes	298 K, 318 K
	10 min	yes	298 K, 318 K
0.66	3–5 min	no	298 K
	3–5 min	yes	298 K, 318 K
	10 min	yes	298 K, 318 K
0.86	3–5 min	no	298 K
	3–5 min	yes	298 K, 318 K
	10 min	yes	298 K, 318 K

### 2.2 Methods

#### 2.2.1 ^1^H and ^23^Na 1D NMR

Standard single pulse experiments were conducted using Avance III HD-NanoBay 300 MHz (^1^H, ^23^Na, 90° pulse), Avance III HD-NanoBay 400 MHz (^23^Na, 30° pulse) and Avance III 500 MHz (^1^H, 30° and 90° pulses) NMR spectrometers (Bruker BioSpin GmbH). All spectra were acquired at 298 ± 1 K and/or 318 ± 1 K, where specified, using Topspin 3.5 pl7 and Topspin 3.6.2 (Bruker BioSpin GmbH) and processed with Topspin 4.0 and/or MestReNova 12.0 (Mestrelab Research S. L.) software. A D_2_O capillary was used for the external lock and as additional internal reference standard for ^1^H spectra. For the ^1^H labeling pattern, see Figure 1. All ^23^Na spectra were referenced against the external solution of 0.1 M NaCl in D_2_O.

#### 2.2.2 ^1^H 2D DOSY NMR

2D Diffusion-Ordered Spectroscopy (DOSY) ^1^H NMR measurements were conducted at 298 ± 1 K and/or 318 ± 1 K, where specified, using Avance III 500 MHz Spectrometer (Bruker BioSpin GmbH). The double-stimulated echo experiment was selected, with gradients ranging from 2.93 to 49.90 G/m in 16 increments [Jerschow and Müller (1996; 1997)]. For each measurement, eight scans were acquired with the diffusion time of 80 ms. All spectra were processed using TopSpin 4.0.7 (Bruker BioSpin GmbH). Within each measurement, all peaks were deconvoluted using TopSpin Dynamic Center 2.6.2 and the diffusion coefficient *D* determined following Eq. 1, with the peak intensity *I*, maximum intensity *I*_0_, gradient length *δ*, gradient distance Δ and gyromagnetic ratio *γ* as a function of the field gradient *x* with a least-squares fit.

$$I=I_{0}*exp\left(-D*\left({\left(2\pi \gamma x\delta \right)}^{2}\right)\left(\frac{\Delta -\delta }{3}\right)10^{4}\right)$$ (1)

#### 2.2.3 Electrochemical impedance spectroscopy

Electrochemical impedance was measured using a potentiostat PGSTAT302N (Metrohm Autolab) with the frequency response analyzer FRA32M. A custom-made experimental setup was used, which consisted of two cylindrical platinum electrodes, placed inside a 5 ml polypropylene measuring cell, additionally sealed to prevent the H_2_O adsorption by EMIM-DCA. Between the series of three consecutive measurements for each sample, electrodes were rinsed with EtOH, milli-Q water and dried at RT.

During the preparation, samples were stirred for minimum 3–5 min to achieve a homogeneous solution and left for 60–120 min to equilibrate, before the electrodes were immersed into the solution. A frequency range of 10–50,000 Hz was measured, using logarithmic points per decade, resulting in 37 data points. All measurements were conducted and impedance spectra were fitted using Nova 2.1.5 software (Metrohm Autolab). Based on the equivalent electrical circuit R(RQ), resistance was obtained for three independently-fitted impedance spectra. The resistance was further recalculated into conductance, with agreement for consecutive measurements within 6% uncertainty.

#### 2.2.4 MD simulation

The initial structures for molecular dynamics (MD) simulations of the ternary IL:water:Na^+^ system were generated using Packmol (Martinez et al. (2009), based on an experimental density of ∼ 1.10 g/ml [Larriba et al. (2014; 2013)]. The length of the cubic box for 100 IL pairs was set to 28 Å and water molecules were distributed randomly in the box according to the different IL:water ratios. Sodium ion will be added to this system after equilibrating the water/IL mixture. Only in one case, a sodium ion was placed at the center of the IL box and water molecules were added in a specified sphere around it. For this case, water molecules were restrained during the heating and cooling stages to keep them close to sodium ion but free to move without any restraint during the simulation. At first, all the simulation boxes were energy minimized. The minimized structure was heated to 228°C in the constant number, volume, and temperature (NVT) ensemble and sequentially cooled down to the target temperature of 25°C in the constant number, pressure, and temperature (NPT) ensemble. The final configuration was then simulated in the NPT ensemble for 20 ns for systems without Na^+^ and 50 ns for a system with Na^+^ while water molecules surrounding it. These equilibrated structures for systems without Na^+^ were considered as an initial structure for the replacement of sodium ion. All these structures were simulated for another 50 ns after Na^+^ addition to water/IL mixtures. The initial structures of EMIM^+^ and DCA^−^ were constructed separately and then optimized with HF/6-31G level of theory in the Gaussian software [Bloino et al. (2009)]. The antechamber program package Wang et al. (2001) with GAFF Wang et al. (2004) generated necessary force field parameters for both EMIM^+^ and DCA^−^ (including bonding, non-bonding, and charge). The partial charges of atoms for anion and cation were calculated from a restrained electrostatic potential (RESP) fit of the isolated ions at the HF/6-31G* level. Since the ionic nature of ILs is largely responsible for their unique properties, the unit charge was scaled down to an absolute value of 0.8 to account for charge transfer and polarizability [Chaban (2011)]. MD simulations were performed by the AmberTools20 program package [Case et al. (2020)]. Additional MD details can be found in ESI.

## 3 Results and discussion

### 3.1 Breakdown of the IL network

As previously reported, the presence of a specific molar fraction of water in EMIM-DCA (*X*_*w*_ = 0.66, corresponding to a molecular ratio 2:1 water:IL) leads to a breakdown of the solvent’s structural arrangement [Dziubinska-Kühn et al. (2021)].

The analysis of ^1^H chemical shifts of water resonances can serve to understand the influence of small-size ions, e.g., NaCl, on the interactions between water and ionic liquid. If the IL self-structuring nano-domains are present, their arrangement influences the hydrogen-bonding (HB) pattern of water molecules inside of the polar nano-domains. As a result, the H/D exchange between H_2_O and D_2_O molecules becomes spatially restricted, and, thus, observable in the NMR timescale. In this case, two separated water species are resolved in ^1^H spectra: pure H_2_O (deshielded) and HOD (shielded) (Supplementary Figure S3) [Dziubinska-Kühn et al. (2021); Saihara et al. (2015)]. The presence of excess water molecules (above the aforementioned *X*_*w*_ = 0.66 threshold), thus leads to a breakdown of the IL structural arrangement, and the H/D exchange is no longer restrained. Consequently, only HOD resonances are visible in ^1^H spectra (Supplementary Figure S2).

In the case of the ternary IL:water:NaCl solution, a low concentration of NaCl (Supplementary Table S3) is selected to assure the full hydration of Na and Cl ions, with a number of water molecules per ion greater than 7. Next, EMIM-DCA is added to hydrated Na^+^ and Cl^−^, based on a systematically decreasing IL:water molecular ratio. The resulting titration curve (Supplementary Figure S3) shows systematically increasing chemical shifts (CS) of proton in H_2_O and HOD, represented as a function of *X*_*water*_. Analogous results obtained for a pure IL-water solution are presented [Dziubinska-Kühn et al. (2021)].

The data shows that, as in the case of a solution without NaCl, the breakdown of the EMIM-DCA nano-domains occurs at *X*_*water*_ = 0.69, corresponding to a molecular ratio 50:100:1 (IL:water:NaCl). This similarity indicates that small quantities of NaCl do not influence the IL-water arrangement inside of the polar and non-polar nano-domains [Aguilera et al. (2015)].

### 3.2 Effect of aggregation on the electrical conductance

The analysis of electrical conductance can bring direct insight into the structure and mobility of the ionic species in the solution [Rosol et al. (2009); Kunze et al. (2011)]. Consequently, optimization of the IL:water:NaCl ternary mixture to obtain the highest conductivity is of high importance for the charge/discharge ratio of the final electrolyte, proving its applicability in battery design [Zhang and Maginn (2015); Gouverneur et al. (2018)].

**FIGURE 2:**
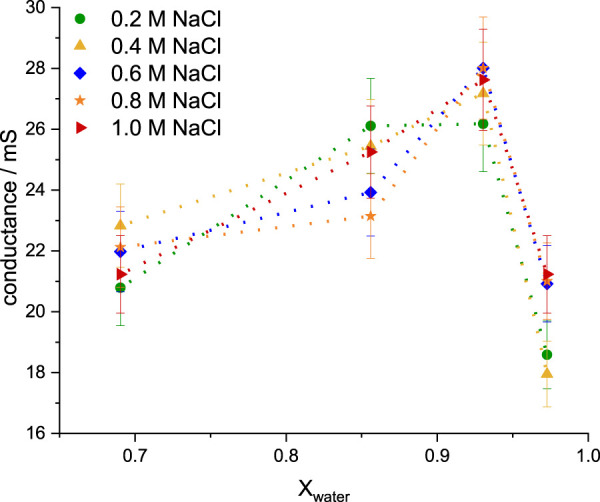
Electrical conductance of ternary solutions containing varying concentrations of NaCl, measured in equilibrated solutions (60–120 min after the preparation) and shown as a function of the molar fraction of water *X*_*water*_: 0.2 M NaCl (green dots), 0.4 M NaCl (yellow triangles), 0.6 M NaCl (blue squares), 0.8 M NaCl (orange stars) and 1.0 M NaCl (red arrows).

If the inorganic salt is added to the ionic liquid in the absence of water, the IL’s conductivity can likely decrease with increasing inorganic salt concentration [Egashira et al. (2009)]. Thus, the presence of water molecules in the IL network is crucial to maintain the high charge density of the salt in the electrolyte, coming from the reduced mutual IL/inorganic salt ion pair formation and having no contribution to the solvent conductance [McEldrew et al. (2021a); Pereiro et al. (2012b)].Hence, electrochemical impedance spectroscopy can be used to analyze the influence of NaCl on the formation of water/IL aggregates upon simultaneous increase of the molar fraction of water and the overall concentration of sodium cations (Figure 2). When EMIM-DCA is added to a solution containing 0.2 M NaCl (≥40 water molecules per Na^+^), the highest conductance is observed at 0.86 ≤ *X*_*w*_ ≤ 0.93, indicating the largest number of free ions present in the ternary mixture, but no significant change in the conductance coming from the increased number of water molecules.

Interestingly, at lower fractions of water and smaller NaCl concentration, the overall conductance is lower than at higher water fraction and medium to high NaCl concentration, even if both maintain a similar water:NaCl ratio. Thus, the formation of ionic liquid species is influenced not only by the presence of second salt, but also the molecular ratio of IL:water:NaCl Becker et al. (2021), similarly to the effect of the type and size of selected IL/inorganic salt cations and anions [Alnashef (2011); Amado-Gonzalez et al. (2017)].

### 3.3 Na^+^ environment in IL-water mixtures

To comprehend the environment of the sodium cation in the IL-water mixtures, an NMR titration study was conducted, using constant number of NaCl molecules with varied IL:water molecular ratio in the solution. In addition, three critical sample compositions are selected, to compare modes of sample preparation: *X*_*w*_ = 0.22, corresponding to different molecular ratio of IL:water:NaCl equal to 60:15:1 (excess EMIM-DCA), and a second ratio of 50:100:1 (*X*_*w*_ = 0.66), representing the maximum water-storage capacity of the IL nano-domains, and *X*_*w*_ = 0.86 (36:215:1), with an excess of water molecules, and thus, no IL aggregation. For each of these solutions, ^1^H and ^23^Na 1D spectra were acquired, using two different mixing orders of the compounds (water + IL + NaCl and water + NaCl + IL), and measured within 1, 6, 12, 24, and 48 h after the sample preparation (see Supplementary Figures S4–S6). Finally, three different mixing protocols were implemented at 298 K and 318 K: short mixing time (3–5 min), with intermediate heating of the sample up to 318 K, and long mixing time (10 min). Details of the sample preparation are described in Table 1.

**FIGURE 3:**
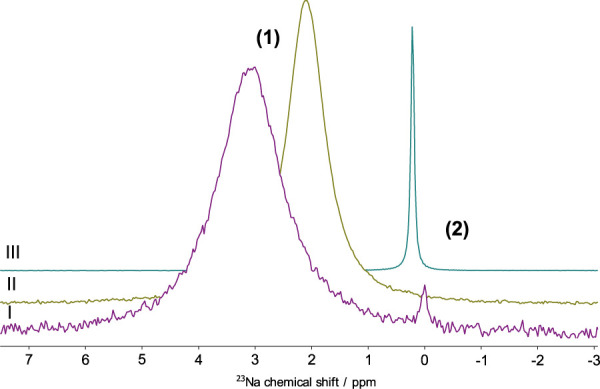
^23^Na NMR resonances acquired using water + NaCl + IL mixing order **(I)**: 1 *μ*M NaCl in 1.24:1 IL:water molecular ratio solution; **(II)**: 0.1 M NaCl solution in 1.24:1 IL:water molecular ratio solution **(III)**: ^23^Na NMR reference standard: 0.1 M NaCl in H_2_O.

Surprisingly, a change in mixing protocols is only observed in the ^23^Na spectra, while ^1^H resonances remain unchanged (Supplementary Figures S7–S12). Peak **(1)** in Figure 3 (CS: 0.64–5.60 ppm) is observed in all spectra and corresponds to the sodium cation surrounded by a mixed solvation shell, consisting of water and IL molecules [Umecky et al. (2013)]. Regardless of the presence of EMIM-DCA nano-domains, upon the addition of water, a systematic shielding of the resonance **(1)** is observed towards the value of 0 ppm, analogous to the ^23^Na reference standard: purely hydrated Na^+^ with low concentration of sodium halide solution [Bloor and Kidd (1968)]. This behaviour confirms the increasing number of water molecules in the nearest environment of the sodium ion, with higher water content [Umecky et al. (2013)].

As shown in Supplementary Figure S4, at low water content, the preparation can be reflected in the chemical shift, and thus, the overall solvent composition. If *X*_*w*_ = 0.22, the CS of **(1)** increases by 1.2 ppm, if longer mixing times and high temperature (HT) are applied. This deshielding effect indicates less water molecules expected to be in the close vicinity of Na^+^. At water content of 0.66 (Supplementary Figure S5), the chemical shift is varying by 0.5 ppm and no significant change is observed at HT. Thus, only at small water fractions, where the competitive IL-water and IL-NaCl interactions are expected, extensive sample preparation (HT and long mixing time) are crucial to achieve an equilibrated environment of the sodium cation.

If the EMIM-DCA nano-domains are not preserved in the presence of excess water molecules (*X*_*w*_ = 0.86), the reversed trend can be observed (Supplementary Figure S6). The application of HT and long mixing time leads to a shielding effect observed for resonance **(1)**, indicating a mixed solvation shell containing more water molecules than before. The observed CS effect is visible also for the FWHM of ^23^Na resonances, but remains unaffected by the mixing order and time of the solution (see Supplementary Figures S13–S15).

Assuming that the IL nano-domains are preserved (*X*_*w*_ = 0.22 and *X*_*w*_ = 0.66), peak **(2)** appears in all spectra acquired for the water + IL + NaCl mixing order, and its CS is molecular-ratio independent and corresponds to the aforementioned pure hydration of Na^+^ at 0 ppm. However, while NaCl is dissolved in water before the addition of EMIM-DCA, the occurrence of **(2)** becomes irregular, regardless of HT, after short mixing times. At X_*w*_ = 0.86, when the excess of water molecules allows for the unrestrained hydration of both, IL and NaCl, two peaks are resolved in the ^23^Na spectra, if long mixing time is applied at RT and HT. Thus, all water molecules are involved in the overall arrangement of the IL-water structure, and only its disruption allows to form isolated hydration shells of the sodium cation, regardless of the mixing order. The analysis of the solution preparation methodology provides an important insight into possible kinetic effects, occurring interdependently on the molecular ratios, mixing order, time and temperature. Introduction of the NaCl molecules to the equilibrated IL-water mixtures leads to the observation of two isolated environments of the sodium cation, remaining stable at HT and for at least 48 h. If NaCl is dissolved in water first and IL is added later, the sodium cation is competing with IL ions for the water molecules to form a solvation shell [Pereiro et al. (2012b); Trindade et al. (2007)]. Consequently, the equilibrated environment of Na^+^ is difficult to be reproduced, due to the intricate kinetics occurring on the short time scale, regardless of the comprehensiveness of the sample preparation.

**FIGURE 4:**
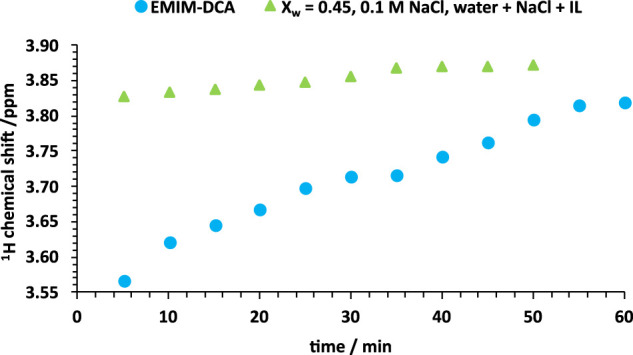
^1^H chemical shifts of water in pure EMIM-DCA and 0.1 M NaCl solution in IL:water mixture (*X*_*w*_ = 0.45) represented as the function of time, acquired at 298 K, after the exposition of the solution to the atmospheric pressure condition.

Figure 4 represents the systematic change of the ^1^H CS for H_2_O resonances, observed as a function of time, when the solution is exposed to the atmospheric pressure. The strongest water absorption is observed during first minutes and gradually decreases over time. In comparison, a solution containing 8.3% of water (*X*_*w*_ = 0.45) shows nearly no water absorption. Thus, if water is added to IL at an early stage of the solution preparation, the hygroscopicity of the solution becomes suppressed and high reproducibility of the results is maintained.

### 3.4 Electrical conductance of water clusters

In addition, the electrical conductance of two solutions, having different mixing orders and constant NaCl concentration, is used to investigate the changes in the ionic species’ formation. Due to the previously observed competitive interaction of IL-water and IL-NaCl pairs, a 10-fold excess of NaCl is used to emphasize the behaviour of Na^+^ and Cl^−^, being the primary charge carriers in the ternary solution. Based on Figure 4, 60–120 min delay the solution preparation and impedance measurements was implemented, to allow the equilibration of ternary mixtures.

**FIGURE 5:**
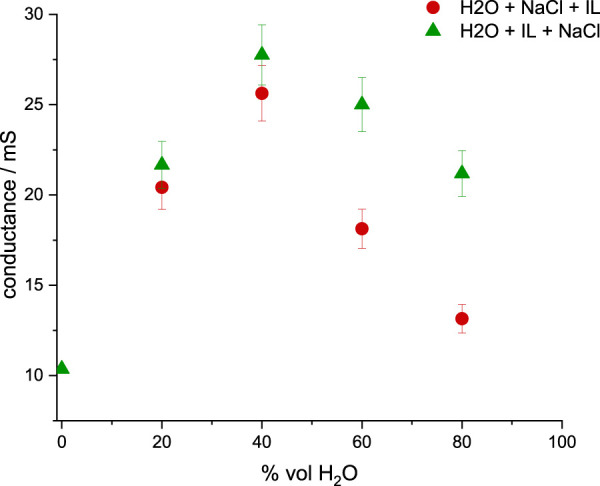
Electrical conductance of IL:water:NaCl ternary solutions, measured in equilibrated solutions (60–120 min after preparation) using varied water + NaCl + IL (red) and water + IL + NaCl (green) mixing orders, 5represented as a function of the molar fraction of water (*X*_*water*_).

As seen in Figure 5, if IL is added before NaCl, a higher conductance is observed, indicating the presence of a larger number of charge carriers in the solution. If IL and water are first mixed, H_2_O/D_2_O molecules will become incorporated into the IL polar domains and no longer be available to hydrate NaCl ions. As previously reported, even in the high vacuum (10^–5^ mbar) conditions, single water molecules remain strongly hydrogen-bonded into the polar domains of EMIM-DCA [Dziubinska-Kühn et al. (2021)]. Consequently, the number of free water molecules available to participate in the Na^+^ hydration is lower and represents the sufficient IL:water:NaCl molecular ratio, enhancing the self-aggregation of water, clustering Na cations.

A similar cluster-seeding phenomenon was already reported in solutions containing small fractions of the inorganic salt added to imidazolium-based ILs [Molinari et al. (2019)]. If inorganic salt and IL share the bulky anion, formation of isolated clusters containing an alkali cation solvated by the anions is observed, leading to a lower conductivity of the solution, due to the overall negative charge of such clusters [McEldrew et al. (2021b)]. In consequence, the excess of the aggregating anions will overcome the positive charge of the alkali cation and a negatively-charged cluster will disturb the charge carrier equilibrium and reduce the charge transfer [McEldrew et al. (2021b); Molinari et al. (2019)].

In this study, sodium halide is selected to avoid the excess of the shared IL/inorganic salt anion. The selected NaCl concentration combined with a large ratio of IL to free water can lead to the formation of sodium-based water clusters, visible with higher conductance of the solution, due to the neutral character of water molecules, not diminishing the role of Na^+^ as a charge carrier. Thus, if the small concentration of NaCl in IL is preserved, simultaneously with large IL:free water ratio, sodium-based water clusters can be formed and observed conductance will increase.

### 3.5 Diffusion coefficients

Additional information about the transport properties in IL-based electrolytes, can come from the diffusion coefficients (*D*) of individual species, observed using Diffusion-Ordered Spectroscopy (DOSY) ^1^H NMR [Mazan and Boltoeva (2017); Zanatta et al. (2019)]. The determined coefficients allow to distinguish two isolated types of aggregates diffusing in EMIM-DCA:water:NaCl mixtures: the EMIM^+^ cation and water-based species.

**FIGURE 6:**
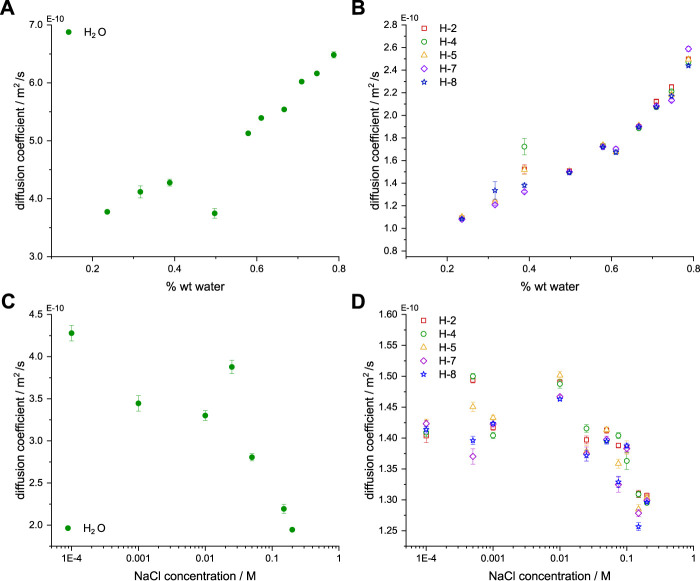
Top: Diffusion coefficients (*D*) of water **(A)** and EMIM^+^ **(B)** in ternary mixtures with constant water:NaCl molecular ratio and shown as a function of the volumetric water content (% wt water). Bottom: Diffusion coefficients of water **(C)** and EMIM^+^ **(D)** in ternary mixtures with constant IL:water molecular ratio and shown as a function of the NaCl concentration (M). All presented *D* correspond to ternary solutions within 1 h from initial mixing of compounds. The time-resolved stability of solutions containing selected IL:water:NaCl molecular ratios is presented in Supplementary Figures S16–S21.

The values of *D* presented in Figures 6A,B are measured upon the addition of EMIM-DCA to a constant 50:1 water:NaCl mixture, where the IL:water molecular ratio is decreasing. The coefficients, determined for water resonances, and thus, representing water-aggregates (Figure 6A), increase from 3.8 e^−10^ m^2^/s for *X*_*w*_ = 0.24 (3.25 EMIM-DCA per 1 water) to 6.5 e^−10^ m^2^/s for *X*_*w*_ = 0.79 (0.25 EMIM-DCA per 1 water). Due to the presence of 50/50 H_2_O/D_2_O mixture, individual coefficients for H_2_O and HOD can be measured, and as previously reported by Shimizu et al., their values do not differ [Saihara et al. (2015)].

Figure 6B shows values of *D* for aromatic and aliphatic protons in the EMIM cation. Results obtained for all the protons are coherent and show a gradual increase from 1.1 e-10 m^2^/s (*X*_*w*_ = 0.24) to 2.50 e^−10^ m^2^/s (*X*_*w*_ = 0.79). Thus, upon the addition of water molecules, both species exhibit the same magnitude of the increasing diffusion (1.7-fold for water and 2.3-fold for EMIM^+^), indicating the mutual relation of decreasing viscosity and increasing ion mobility, frequently observed in IL-water binary systems [Bayles et al. (2019); Yaghini et al. (2014); Liu X. et al. (2011)].

Surprisingly, at *X*_*w*_ = 0.69, when the breakdown of the IL nano-domains is expected, no change in the diffusion coefficients’ slope is notable, implying that the gradual dissolution of bulky EMIM^+^ aggregates is disrupted by the systematically growing water-based aggregates. An analogous observation was previously reported for pure EMIM-DCA and water mixtures, where a transition region, representing the defragmentation of IL nano-domains was identified at 0.66 < *X*_*w*_ ≤ 0.91 [Dziubinska-Kühn et al. (2021)]. Thus, similarly to the observed ^1^H NMR CS behaviour (Supplementary Figures S6–S11), the presence of a small amount of inorganic salt does not influence the principal EMIM-DCA and water interaction.

Furthermore, as seen in Figures 6C,D, the diffusion coefficients were also measured in a series of ternary mixtures, containing constant IL:water molecular ratio and water:NaCl ratio varying from 0.1 mM to 0.2 M NaCl. Upon the increasing number of NaCl dissolved ions, the value of *D* coefficient decreases only by a factor of 2.2, from 4.3 e^−10^ m^2^/s to 1.9 e^−10^ m^2^/s, indicating the strong influence of alkali metal cation and halide anion on the water-based species formed in the IL nano-domains. A similar type of behaviour was previously observed in the presence of low NaCl concentrations (∼0.1 mM - 0.1 M) [Burkell and Spinks (1951)]. As presented in Supplementary Figures S16–S21, the time-dependent analysis of the selected diffusion coefficients, performed at 298 K and 318 K, confirmed their high stability within the constant IL:water:NaCl molecular ratio, but also regardless of the mixing order during the ternary solution preparation.

The general diffusivity dependence on the halides concentration is considered to have a non-linear character and at medium-to-high NaCl concentrations diffusion coefficients will increase again [Vitagliano and Lyons (1956)]. Moreover, at low NaCl concentrations, two distinguishable water-based species can be observed, using diffusion coefficients: NaCl-aggregated and water-diluted regions, containing single inorganic salt ions [Hayamizu et al. (2021); Georgalis et al. (2000)]. Thus, larger diffusion coefficients, measured at the higher water:NaCl ratio, indicate a smaller size of water-based species, due to the extensive HB network formed by water molecules solvating the inorganic salt ions [Tielrooij et al. (2010)]. Interestingly, the behaviour of alkali metal halides in the water solution is being widely discussed, due to their ability to substitute water molecules inside of the percolated solvent network [Ding et al. (2014)]. Moreover, rigidity of the formed hydration shell depends on the aforementioned HB network formation, resulting from the strength of the alkali metal cation and/or halide anion hydration [Tielrooij et al. (2010)]. The presence of isolated water-clusters based on the Na^+^ node is additionally observable in the ^23^Na NMR spectra (Figure 3), where two resonances are well-resolved in the spectrum acquired in 1 *μ*M NaCl solution. The occurrence of the second peak at CS = 0 ppm indicates the presence of a fully isolated water environment of sodium cation [Bloor and Kidd (1968)].

The diffusion coefficients of protons in the EMIM cation coherently show a plateau at 1.45 e^−10^ m^2^/s between 0.1 mM and 0.05 M NaCl concentration of the EMIM-water solution and afterwards slightly decrease to 1.35 e^−10^ m^2^/s at 0.1–0.2 M Nacl concentration. Thus, the number of NaCl molecules has little effect on the behaviour of IL-based aggregates in the presence of medium NaCl concentration. Interestingly, only a single, broad resonance **(1)** can be observed in ^23^Na NMR spectrum acquired in 0.1 M NaCl solution (Figure 3). Moreover, the CS of the detected peak is decreased, in comparison with the broad peak **(1)** in 1 *μ*M solution. Therefore, the presence of a larger amount of inorganic salt, leads to mixed NaCl-water aggregates inside of the IL nano-domains, whereas at low NaCl concentrations two separated species are detected and less water molecules participate in the sodium hydration.

### 3.6 Water/IL simulations

To obtain further insight into the structural arrangement of the ternary mixture (IL:water:Na^+^) and to investigate the impact of the mixing order on the environment of the Na^+^ in the initial structure, two different simulation protocols were designed. Four different systems with varying IL:water molecular ratios were simulated in which Na^+^ was added to a fully pre-equilibrated water/IL mixture. They will be further on referred to as W2, W5, W10, and W40 for IL:water molecular ratios of 50:1, 20:1, 10:1, and 60:40, respectively. Furthermore, an additional simulation was performed for W5 with a 20:1 molecular ratio (IL:water), in which water molecules were added close to the Na^+^ in the IL solution instead of a random distribution of water (W5F).

**FIGURE 7:**
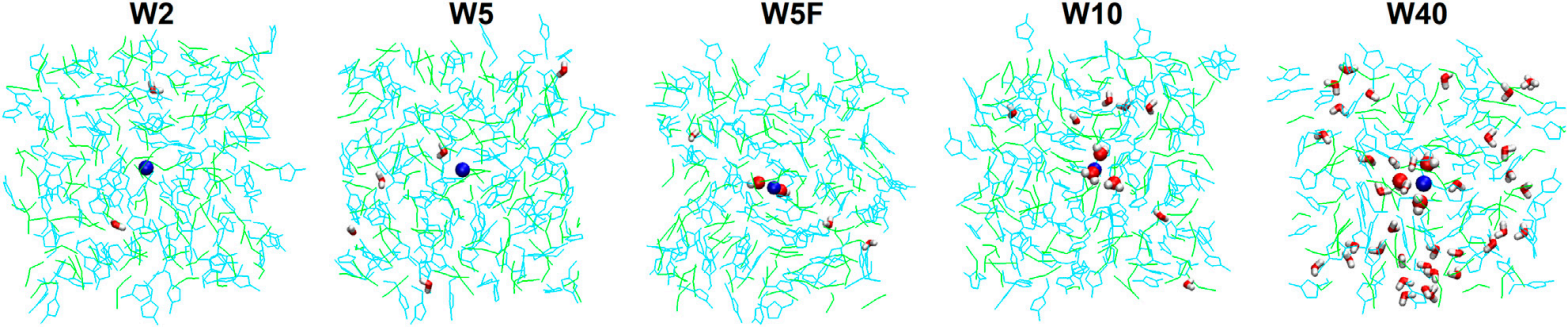
Snapshots of the simulated systems including W2, W5, W5F, W10, and W40. Water molecules in the sodium hydration shell and sodium cation are shown by the sphere model while EMIM^+^ and DCA^−^ are represented by line models with light blue and green colors, respectively. The color code for atoms is sodium: blue, oxygen: red, and hydrogen: white.

The MD trajectories indicate the arrangement of water molecules between IL cations and anions around the sodium ion, a representative snapshot for each system is shown in Figure 7. The four systems with the initial random distribution of water molecules in the IL network and addition of Na^+^ to water/IL exhibit two different behaviours after simulation: 1) water molecules distribute outside of the sodium solvation shell and also outside of the non-polar nano-domain of IL in low water content systems (W2 and W5), 2) a fraction of water molecules attains the first solvation shell of the sodium ion, while others remain between IL molecules in higher water content systems (W10 and W40). Therefore, the sodium ion experiences two different environments, concerning the water molecules, induced by varying IL:water ratio. Interestingly, for W5F, 0–2 water molecules stay in the solvation shell around the sodium ion over the entire simulation time while the attendance of water in Na^+^ solvation shell is very rarely for W5. Thus, water molecules and DCA anions can exchange their position in the solvation shell constantly in comparison to the non-restrained random distributed W5 system with the same water content. When water molecules completely surround the sodium ion before adding IL molecules to the system, this equilibrium shifts towards the higher affinity for water molecules. The presence of water molecules close to the sodium ion before starting the equilibrating simulation was intended to produce an initial solvation shell similar to an initial dissolvation of sodium ion in water. Consequently, the comparatively short time of the simulation can mimic the long-term kinetics of water solvation observed in DOSY NMR.

**FIGURE 8:**
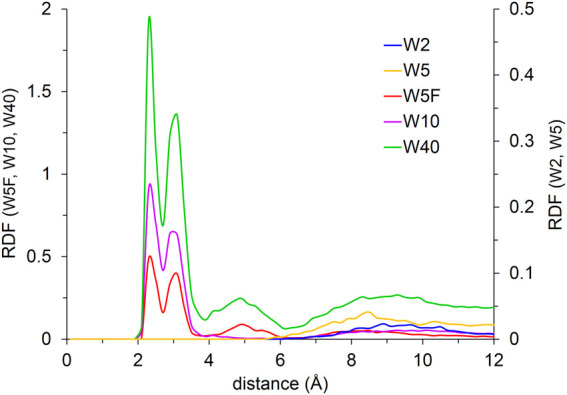
RDF for water molecules with respect to Na^+^. W2 and W5 systems are presented on the right axis, systems of W5F, W10, and W40 are presented on the left axis. In this work, the RDFs are scaled by appropriate number densities to facilitate the comparison across systems with different water/IL ratios.

To quantify the aforementioned observations, the structural arrangement of the simulated systems is additionally investigated by calculating the radial distribution functions (RDFs) over the last 20 ns of the simulation, and the coordination number *via* integration of the first peak of the corresponding RDF. The RDF based on the distance between the oxygen atom of water molecules and sodium ion characterizes the spatial coordination of water molecules around the sodium ion (Figure 8).

**FIGURE 9:**
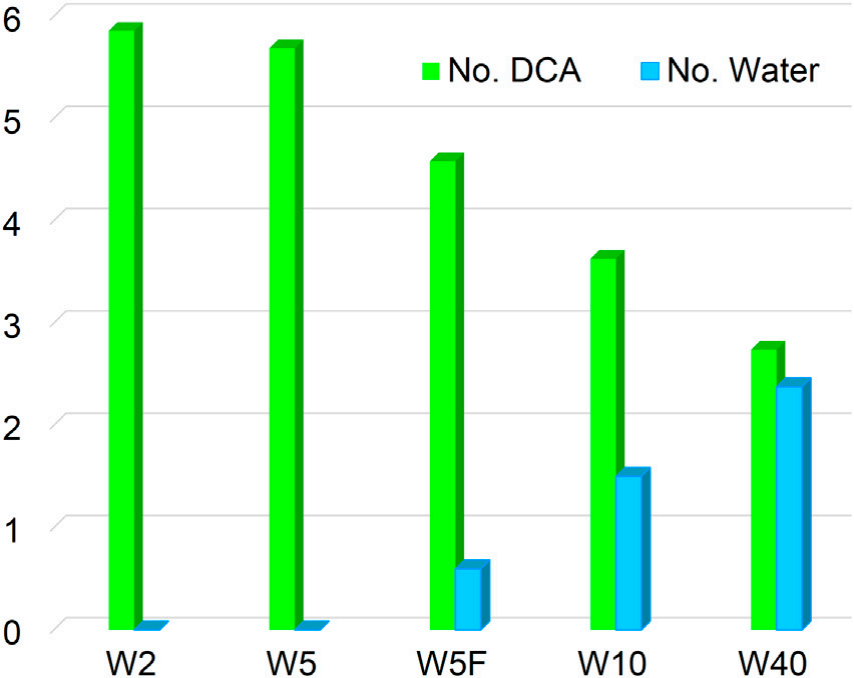
The coordination number of water molecules and DCA anions around Na^+^.

The two systems with fewer water molecules (W2 and W5) show a very short broad peak at distances above 6 Å, demonstrating water molecules bind preferentially to IL molecules inside of the polar nano-domain instead of participating in the sodium ion hydration shell. According to anion-Na^+^ RDF, (Supplementary Figure S22), DCA anions form the first shell around the sodium ion, while the water molecules do not incorporate into the first solvation shell of the sodium ion. Also, the average coordination number of water molecules around sodium ions is almost equal to zero for W2 and W5, as shown in Figure 9.

A well-structured first peak at 2 to 4 Å and a shoulder between 4 and 10 Å for two systems with high water content (W10 and W40) are observed in Figure 8. A fraction of water molecules migrates from the IL network into the sodium hydration shell, forming two uneven distinct RDF peaks near Na^+^, while others, distributed into IL, form shoulder. Therefore, the environment of the sodium ion changes with increasing water content, and the first hydration shell is comprised of both DCA anions and water molecules. Consequently, the binding sites in the first solvation shell are competitively occupied and strongly influenced by the availability of water. The RDFs also show that additional water molecules get more structured when the water content increases, until the aforementioned nano-domains breaking point. The distribution of water molecules into IL can be interpreted as a formation of a network of HB. Water molecules form inter- and intra-hydrogen bonds with DCA anions or the acidic proton in the imidazolium aromatic ring and themselves, respectively. It was shown that the presence of water molecules considerably impacts cation − anion ion pair interaction through HB. The HB-based networks around cations and anions alter with the addition of water but do not change directly proportional to water content [Huang et al. (2017)]. Water molecules form small clusters at low concentrations and assemble into a continuous network at high concentrations. Small anions can disrupt these networks by breaking water-water HB while other larger anions form HB with water directly [Liu H. et al. (2011); Hanke and Lynden-Bell (2003)], showing the dominant role of an anion structure on the interaction between water and IL [Khan et al. (2014); Jiang et al. (2021)]. The formation of HB between water molecules and DCA anions is shown in Supplementary Figure S23. The behaviour of RDF for W5F differs from W5 and shows two uneven peaks at 2 to 4 Å, similar to results at a higher water content, indicating a mixed solvation shell (see also coordination number in Figure 9). The discrepancy between these two systems leads to the availability of water for Na^+^ in W5F. This indicates that the sodium hydration shell in ILs not only depends on water concentration but can be also disturbed by the mixing order of contents.

## 4 Summary and conclusion

This study focuses on the influence of alkali metal halides, specifically NaCl, on the behaviour of water in EMIM-DCA nano-domains. Firstly, ^1^H NMR spectra revealed that at medium-to-low NaCl concentration, no change in the IL nano-domains is observed. Consequently, the molar fraction of water required to cause the disruption of EMIM-DCA network into random aggregates equals *X*_*w*_ = 0.69 and stays in a good agreement with previously reported *X*_*w*_ = 0.66 for pure EMIM-DCA-water binary system.

In addition, a large influence on the structural arrangement of the ionic species in the EMIM-DCA–water–NaCl ternary system was revealed by changing the mixing order of compounds at constant molecular ratios, especially in the presence of the IL nano-domains. The acquired ^23^Na NMR resonances, as well as impedance spectra, showed the presence of sodium cations, becoming a node for hydrating water molecules, forming a well-isolated environment. The occurrence of those aggregates was only observed if IL and water were mixed before the addition of NaCl and/or selected IL:water:NaCl ratio. Hence, water molecules situated in the IL nano-domains can rearrange their position if a strong salt is added. This effect was confirmed with DOSY NMR, where the diffusion coefficients were independently influenced by both: EMIM:water and water:NaCl ratios.

The environment of Na^+^ in molecular dynamics simulations showed different compositions: from pure DCA^−^ in low water content to a combination of water and IL in high water content. The first solvation shell of Na^+^ depends on the IL:water molecular ratio. Most water molecules remain between IL networks in higher water concentrations and a lower fraction migrates to the hydration shell of the Na^+^. Changing the Na^+^ environment from water/IL to pure water in the initial structure can affect the hydration shell of the sodium ion and preserves a small fraction of water molecules.

The presented results demonstrate that the method of the electrolytes processing is highly relevant for the IL - water - inorganic salts battery applications. Without a doubt, in the selected ternary mixture, the IL–water interactions are stronger than water–NaCl and IL–NaCl interactions. Furthermore, the order in which the compounds are mixed and their molecular ratio are revealed to be the crucial elements of solution, tuning the environment of sodium cations.

As previously reported, the combination of sodium halides and short side-alkyl chain imidazolium-based ILs can lead to the formation of suitable candidates for energy-storage electrolytes [Sun et al. (2019); Bloino et al. (2009)]. The presented novelty of selecting neat IL, proven to form segregated nano-domains in the presence of water (Dziubinska-Kühn et al. (2021); Saihara et al. (2015), brings a possible new class of electrolytes with their overall properties determined by the IL intermolecular arrangement and conductive behaviour tuned by the moderation of water:NaCl molecular ratio within the IL network. Thus, simultaneously preserved peculiar properties of ionic liquids and their energy-storage applications are recommended to be verified for further EMIM-based ILs.

## Data Availability

The raw data supporting the conclusions of this article will be made available by the authors, without undue reservation.
