# Supplementary material for: Influence of alkali metals on water dynamics inside imidazolium-based ionic liquid nano-domains
Source: Front Chem. 2022 Nov 15;10:1028912. doi: 10.3389/fchem.2022.1028912 (PMC9707627; doi:10.3389/fchem.2022.1028912)
Supplement: Supplementary file 1 [file DataSheet1.pdf]

Supplementary Material

1 MD SIMULATION DETAILS

Periodic boundary conditions were assigned in the XYZ direction. SHAKE algorithm was used to constrain all bonds including hydrogen atoms (J. Comput. Phys. 23, 327–341 (1977)). The particle mesh Ewald (PME) method was applied for the treatment of electrostatic interactions (J. Chem. Phys. 98, 10089–10092 (1993)). A cutoff of 10 Å was used for van der Waals and electrostatic interactions. A 1 fs time step was set to integrate the equation of motion. The temperature and pressure were controlled by the Langevin temperature coupling scheme (J. Chem. Phys. 72(4), 2384–2393 (1980)) with collision frequency (gamma_ln) of 5.0 ps^{-1} and isotropic position scaling protocol (J. Comput. Phys. 23(3), 327–341 (1977)) with pressure relaxation time (taup) of 2 ps, respectively.

2 EQUILIBRATION PROTOCOLS

For both kinds of IL:water:Na^+ arrangements, without sodium ion at the first time and with sodium ion while water molecules surround it, the equilibration process is similar after constructing the initial structures. 25,000 steps of conjugate gradient followed by 25,000 steps of steepest descent were utilized to energy minimize the simulation box. The minimized structure was heated to 228 °C in an NVT ensemble for 500 ps to achieve thorough mixing of the ions and to eliminate any artifacts in the initial structure. Then, the temperature was decreased in two steps of 100 °C down to 25 °C, each run for 200 ps. For a system with sodium which was fully solvated by water molecules, water molecules were restrained during the heating and cooling stages to keep them close to sodium ion while there was not any restraint for systems without sodium ion. Water molecules were restrained with a 20 kcal/(mol.$Å^2$) force constant during the heating and cooling stages to keep water molecules in a specific radius around the Na^+ for a system with the sodium ion. The resulting structures after the final cooling stage were simulated for 20 ns for systems without sodium ion and 50 ns for a system with the solvated sodium ion. For a system with a fully solvated sodium ion, the first 30 ns were considered as equilibration and the last 20 ns as a production run. The profile of densities for all systems except W5F after the 20 ns equilibration is shown in Fig. S-1A. For the systems without sodium ion, a structure was extracted from the MD trajectory and then sodium ion was randomly added to the box of IL:water. Finally, a 50 ns simulation was performed after sodium addition and the first 30 ns was considered as equilibrating time and the final 20 ns for analyzing. The trend of density and potential energy as a function of time for these four systems (W2, W5, W10, and W40) in addition to W5F are shown in Fig. S-1B-C. After the end of the simulation, the trajectories were rearranged such that Na^+ be placed at the center of the box.

Table S1. The molar fraction and volumetric fraction of water (50/50 v/v $H_2O + D_2O$), quantities of used water and EMIM-DCA, at constant NaCl concentration of 0.1 M (calculated for total volume of EMIM-DCA + water) in selected samples, analyzed using ^{1}H and ^{23}Na 1D NMR at 298 K.

$X_{H_2O+D_2O}$	Water (% vol)	Water volume (μL)	EMIM-DCA volume (μL)
0.19	2.5	15.0	585.0
0.32	5.0	30.0	570.0
0.42	7.5	45.0	555.0
0.50	10.0	60.0	540.0
0.56	12.5	75.0	525.0
0.61	15.0	90.0	510.0
0.65	17.5	105.0	495.0
0.69	20.0	120.0	480.0
0.79	30.0	180.0	420.0
0.86	40.0	240.0	360.0
0.90	50.0	300.0	300.0

Table S2. The molar fraction of water (50/50 v/v $H_2O + D_2O$) and the ratio of EMIM-DCA and water molecules in selected samples, at constant 1.0:0.022 water:NaCl molecular ratio, analyzed using ^{1}H 2D DOSY NMR at 298 K.

$X_{H_2O+D_2O}$	NO. (EMIM-DCA)	NO. (water)
0.67	0.50	1.0
0.64	0.56	1.0
0.61	0.64	1.0
0.58	0.73	1.0
0.50	1.01	1.0
0.39	1.58	1.0
0.32	2.16	1.0
0.24	3.24	1.0

Table S3. The molar fraction of water (50/50 v/v $H_2O + D_2O$) and the ratio of water and NaCl molecules in the selected samples, at constant 1.24:1.0 EMIM-DCA:water molecular ratio, analyzed using 1H 2D DOSY NMR at 298 K.

$X_{H_2O+D_2O}$	NO. (water)	NO. (NaCl)
0.45	1.0	0.108
0.45	1.0	0.054
0.45	1.0	0.043
0.45	1.0	0.032
0.45	1.0	0.022
0.45	1.0	0.011
0.45	1.0	0.005
0.45	1.0	0.002

Table S4. The volumetric fraction of EMIM-DCA and water (50/50 v/v H_2O + D_2O), number of EMIM-DCA molecules per one water molecule and number of water molecules per one NaCl molecule in selected samples, analyzed using electrochemical impedance spectroscopy at 298 K.

EMIM-DCA (% vol)	Water (% vol)	NaCl concentration	NO. (water/EMIM-DCA)	NO. (water/NaCl)
0.20	0.80	0.2 M	35.7	227.8
0.20	0.80	0.4 M	35.7	113.9
0.20	0.80	0.6 M	35.7	75.9
0.20	0.80	0.8 M	35.7	56.9
0.20	0.80	1.0 M	35.7	45.6
0.40	0.60	0.2 M	13.4	178.6
0.40	0.60	0.4 M	13.4	89.3
0.40	0.60	0.6 M	13.4	59.5
0.40	0.60	0.8 M	13.4	44.6
0.40	0.60	1.0 M	13.4	35.7
0.60	0.40	0.2 M	5.9	129.4
0.60	0.40	0.4 M	5.9	64.7
0.60	0.40	0.6 M	5.9	43.1
0.60	0.40	0.8 M	5.9	32.3
0.60	0.40	1.0 M	5.9	25.9
0.80	0.20	0.2 M	2.2	80.2
0.80	0.20	0.4 M	2.2	40.1
0.80	0.20	0.6 M	2.2	26.7
0.80	0.20	0.8 M	2.2	20.1
0.80	0.20	1.0 M	2.2	16.0

Table S5. Partial charge based on RESP method and non-bonded parameters (sigma and epsilon) on each atom of EMIM cation and DCA anion which was used in this work. A charge scale of 0.8 was employed on the unit charge.

Atom	Charge	σ (Å)	ε (kJ/mol)
$EMIM^+$			
CR	-0.0336	3.400	0.3598
NA(m)	0.1936	3.250	0.7113
NA(a)	0.0128	3.250	0.7113
CW(m)	-0.2199	3.400	0.3598
CW(a)	-0.2016	3.400	0.3598
H5	0.2440	1.782	0.0628
H4(m)	0.2609	2.511	0.0628
H4(a)	0.2711	2.511	0.0628
CT(m)	-0.3357	3.400	0.4577
H1	0.1701	2.471	0.0657
CT(a1)	0.0461	3.400	0.4577
H1	0.0885	2.471	0.0657
CT(a2)	-0.1404	3.400	0.4577
HC	0.0718	2.650	0.0657
DCA^-			
N1	-0.8523	3.250	0.7113
N2	-0.8223	3.250	0.7113
N3	-0.8223	3.250	0.7113
C1	0.7485	3.399	0.8786
C2	0.7485	3.399	0.8786

Table S6. Force field bonding parameters for EMIM and DCA ions used in this work.

bonds	r_0 ($\AA$)	K_r (kJ/mol.$\AA^2$))
	$EMIM^+$	
CT-CT	1.526	1297
CT-H1	1.080	1423
CT-HC	1.090	1423
CT-NA	1.472	1172
CR-H5	1.070	1590
CR-NA	1.325	1674
CW-H4	1.070	1611
CW-NA	1.378	1506
CW-CW	1.343	1715
	DCA^-	
N1-C1	1.202	3314
N1-C2	1.202	3314
N2-C2	1.153	3994
N3-C1	1.153	3994

angles	θ_0 (deg)	K_θ (kJ/mol.rad^2))
	$EMIM^+$	
CT-CT-H1	109.5	159
CT-CT-HC	109.5	155
CT-CT-NA	112.2	293
H1-CT-H1	109.5	146
HC-CT-HC	109.5	142
H1-CT-NA	109.5	230
CW-NA-CT	125.7	209
CR-NA-CT	126.3	209
CW-CW-NA	107.1	502
CR-NA-CW	108.0	502
NA-CR-NA	109.9	502
H4-CW-NA	122.1	126
H5-CR-NA	125.7	126
CW-CW-H4	130.7	126
	DCA^-	
N1-C1-N3	174.0	271
C1-N1-C2	140.0	275

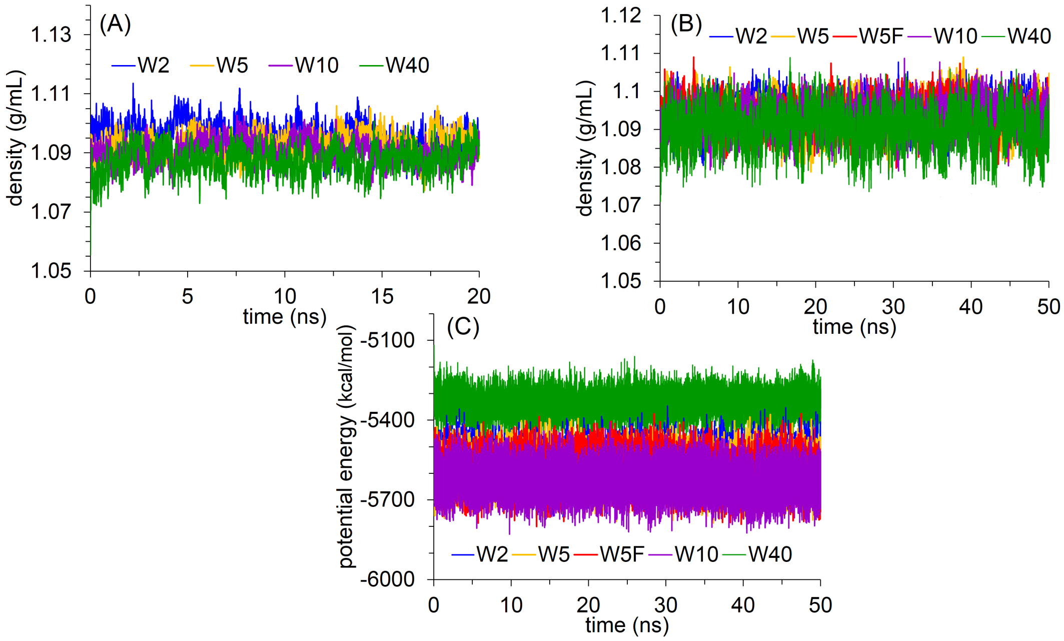

Figure S1. (**A**) The density profile as a function of time for different systems at the first equilibration stage. The trend of (**B**) density and (**C**) potential energy as a function of time for all studied systems for the last 50 ns of simulation.

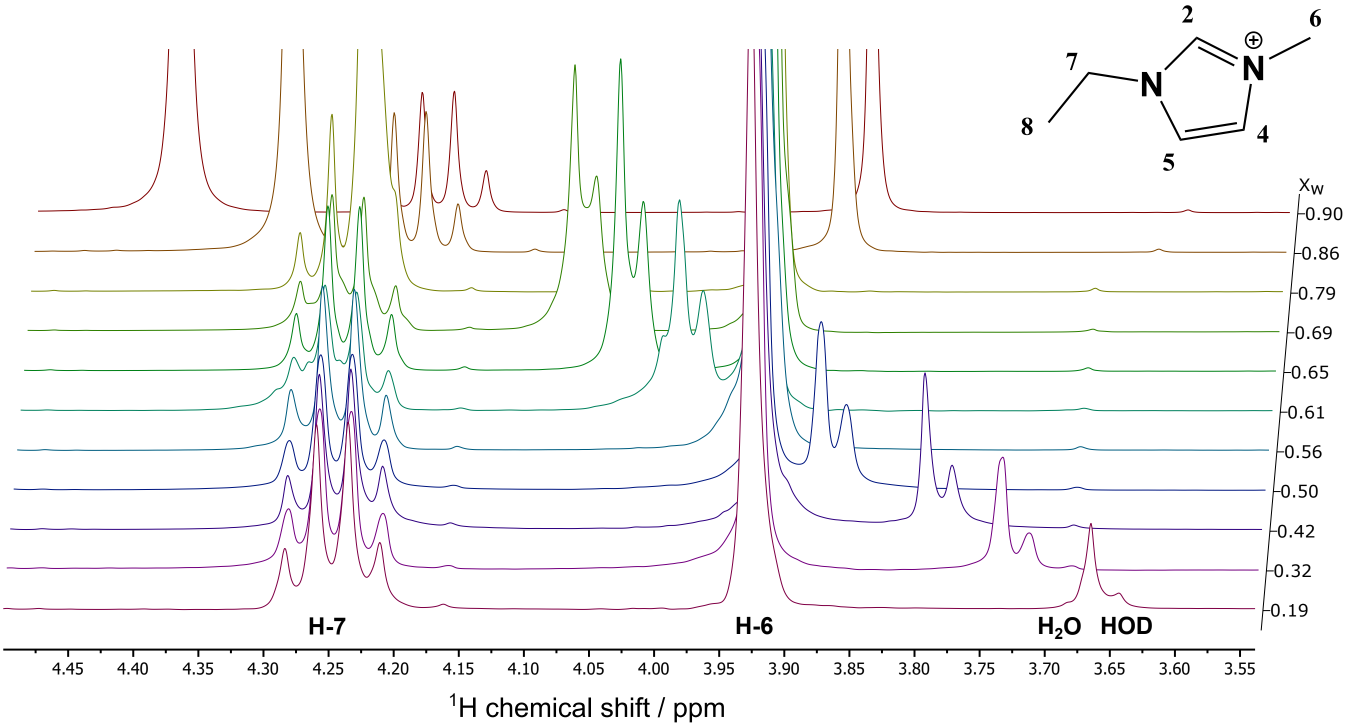

Figure S2. ^{1}H 1D NMR (inset) obtained at 300 MHz, showing the gradual change of the chemical shift and intensity of H_2O (deshielded) and HOD (shielded) peaks in the spectra between $0.19 \leq X_{water} \leq 0.90$ and containing constant 0.1 M NaCl, measured at 298 K. The labeling pattern of the EMIM cation is shown in the upper right corner.

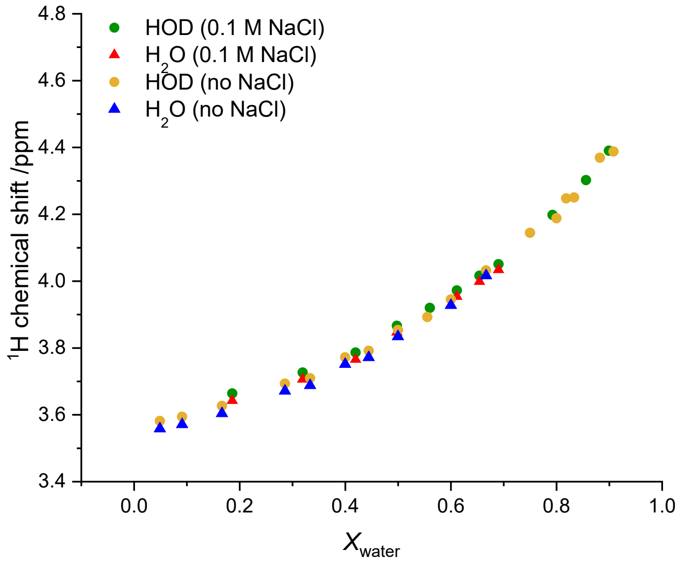

Figure S3. ^{1}H 1D NMR results for EMIM-DCA – water – NaCl ternary mixture containing 50/50 v/v H_2O/D_2O and constant 0.1 M NaCl concentration; chemical shifts of the water resonances: H_2O (green dots) and HOD (red triangles), shown as a function of the molar fraction of water X_{water}. For comparison, ^{1}H chemical shifts of water resonanc-es in the absence of NaCl are added: H_2O (yellow dots) and HOD (blue triangles). All spectra were acquired at 298 K.

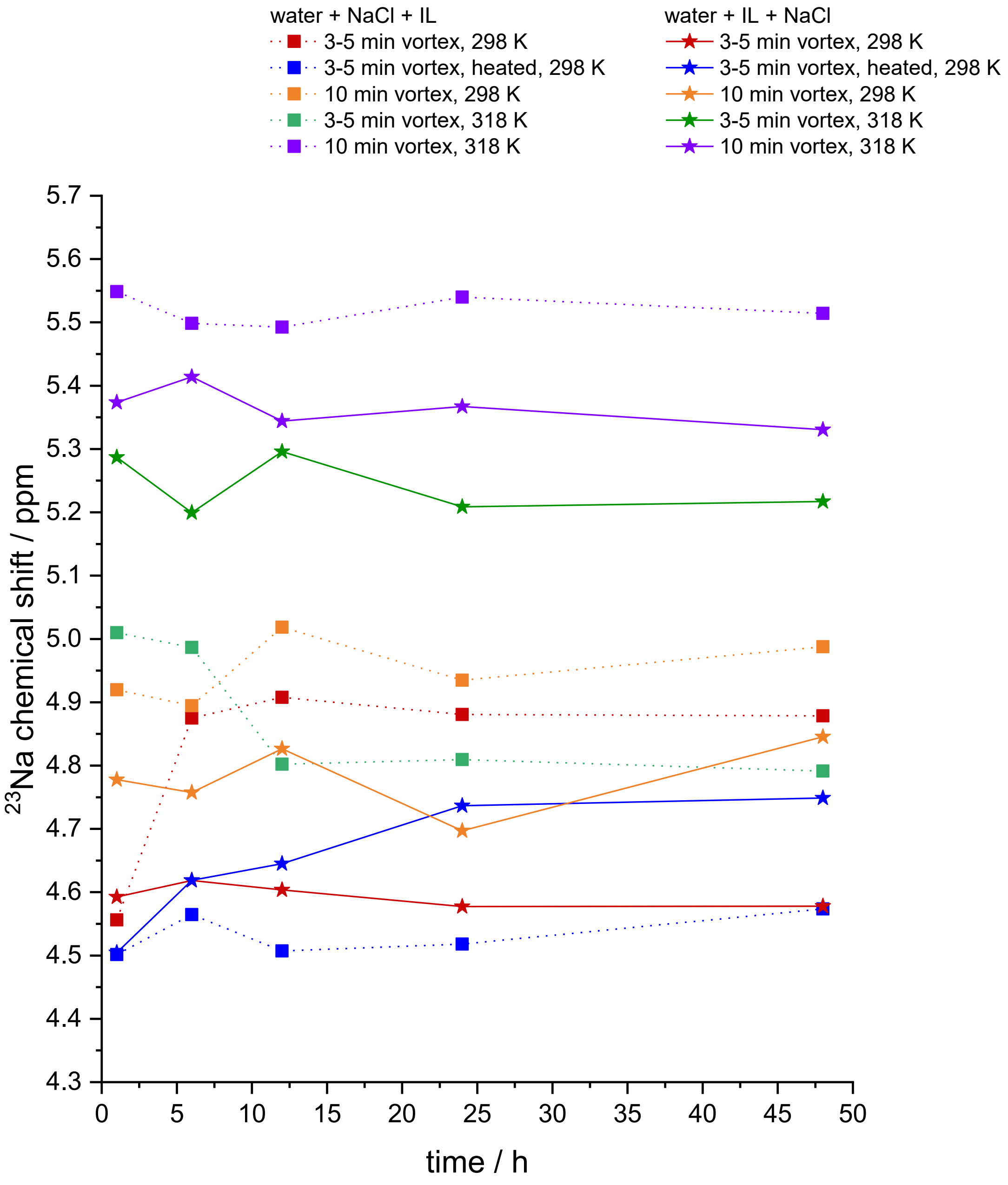

Figure S4. Comparison of ^{23}Na chemical shifts (CS) of peak (**1**), measured in the EMIM-DCA – water – NaCl ternary solutions (X_w = 0.22, 0.1 M NaCl) for different protocols applied during the sample preparation: varying mixing time (3 - 5 min or 10 min), heating of the solution to 318 K, applied before (and not during) the measurement ("heated"), and temperature (measured at 298 K or 318 K). Two mixing orders of the compounds are used: water + NaCl + IL (square) and water + IL + NaCl (star). The CS are presented as the function of time, representing the age of the sample, selected between 1 h and 48 h.

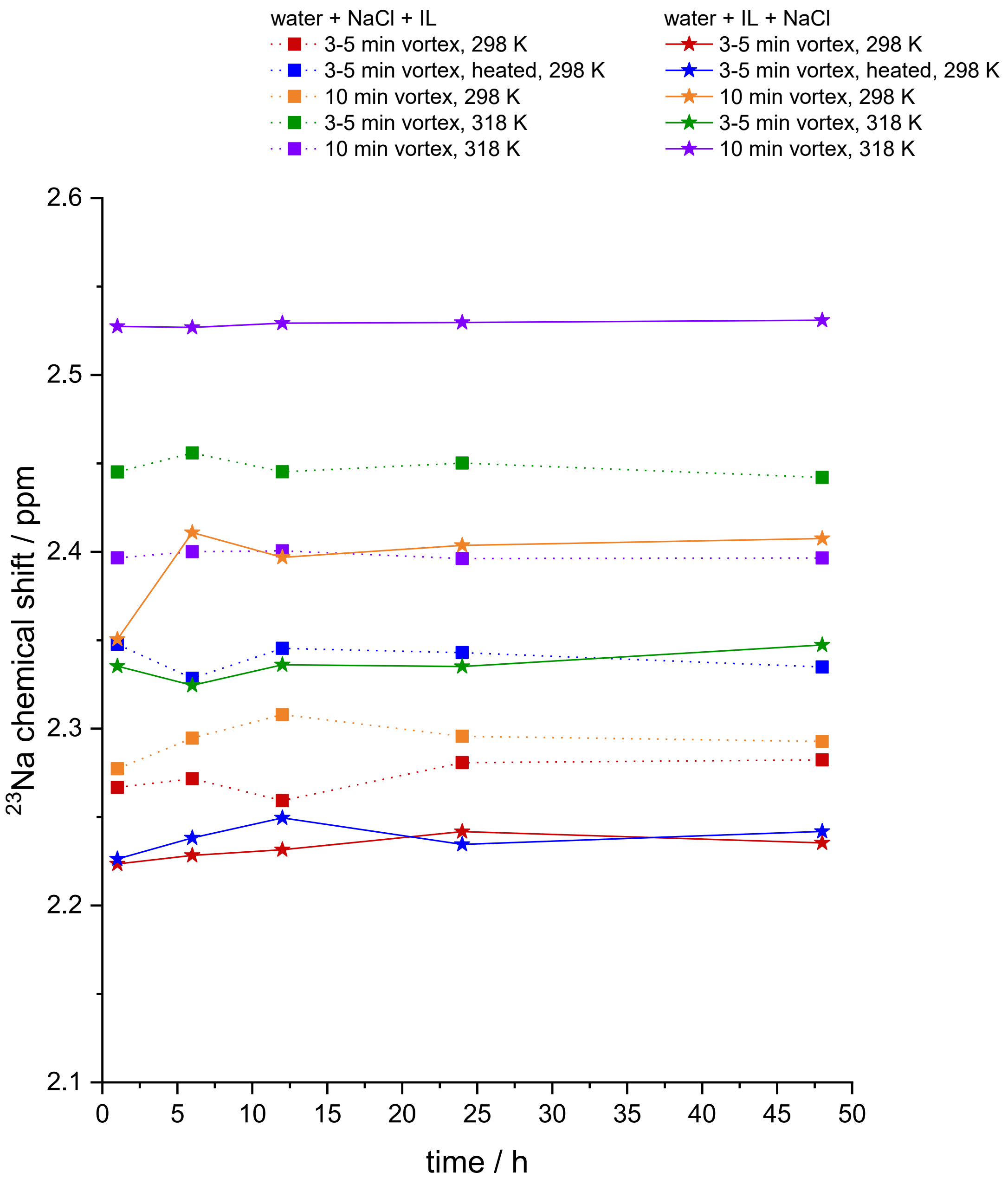

Figure S5. Comparison of ^{23}Na chemical shifts (CS) of peak (**1**), measured in the EMIM-DCA – water – NaCl ternary solutions (X_w = 0.66, 0.1 M NaCl) for different protocols applied during the sample preparation: varying mixing time (3 - 5 min or 10 min), heating of the solution to 318 K, applied before (and not during) the measurement ("heated"), and temperature (measured at 298 K or 318 K). Two mixing orders of the compounds are used: water + NaCl + IL (square) and water + IL + NaCl (star). The CS are presented as the function of time, representing the age of the sample, selected between 1 h and 48 h.

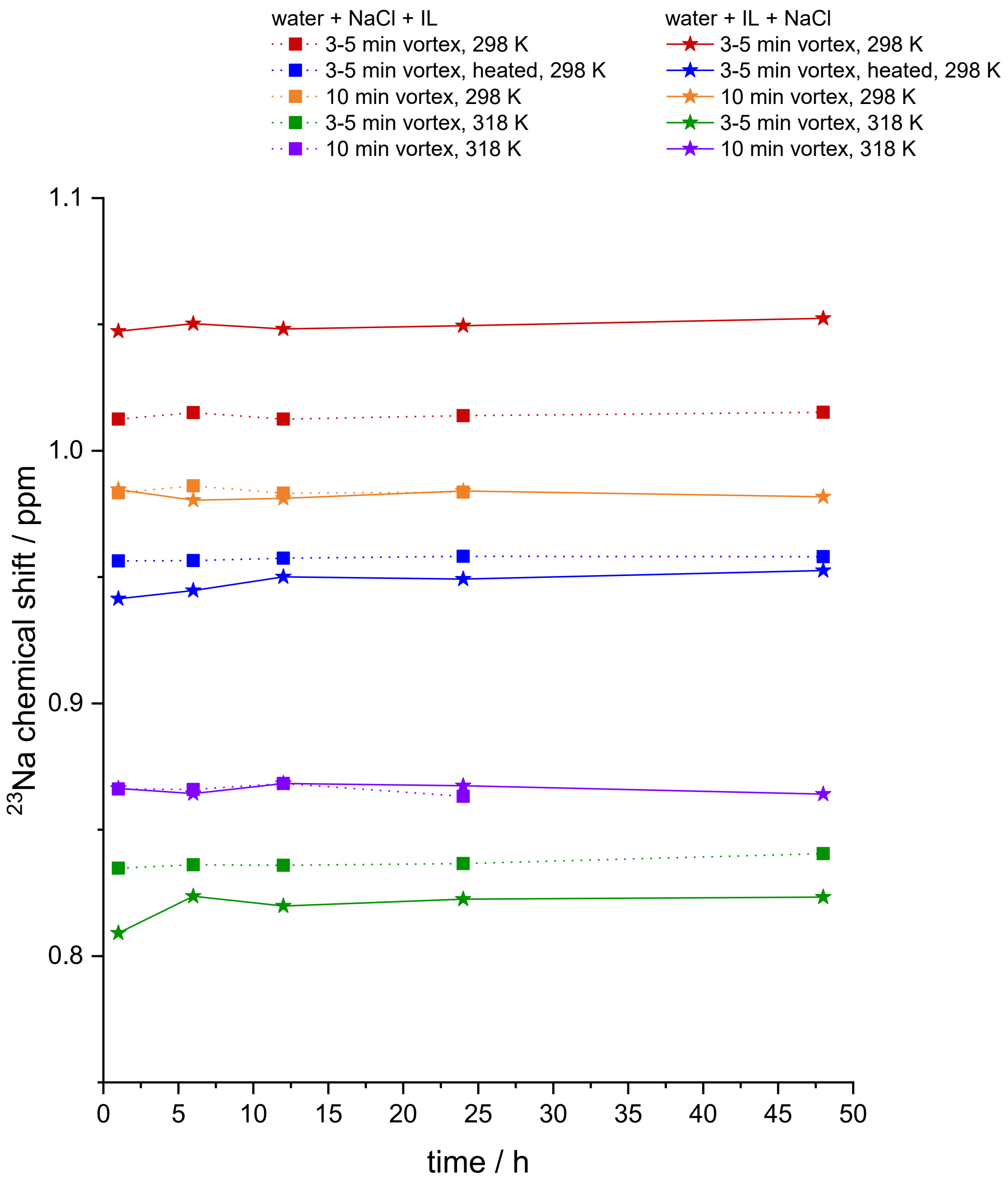

Figure S6. Comparison of ^{23}Na chemical shifts (CS) of peak (**1**), measured in the EMIM-DCA – water – NaCl ternary solutions (X_w = 0.86, 0.1 M NaCl) for different protocols applied during the sample preparation: varying mixing time (3 - 5 min or 10 min), heating of the solution to 318 K, applied before (and not during) the measurement (“heated”), and temperature (measured at 298 K or 318 K). Two mixing orders of the compounds are used: water + NaCl + IL (square) and water + IL + NaCl (star). The CS are presented as the function of time, representing the age of the sample, selected between 1 h and 48 h.

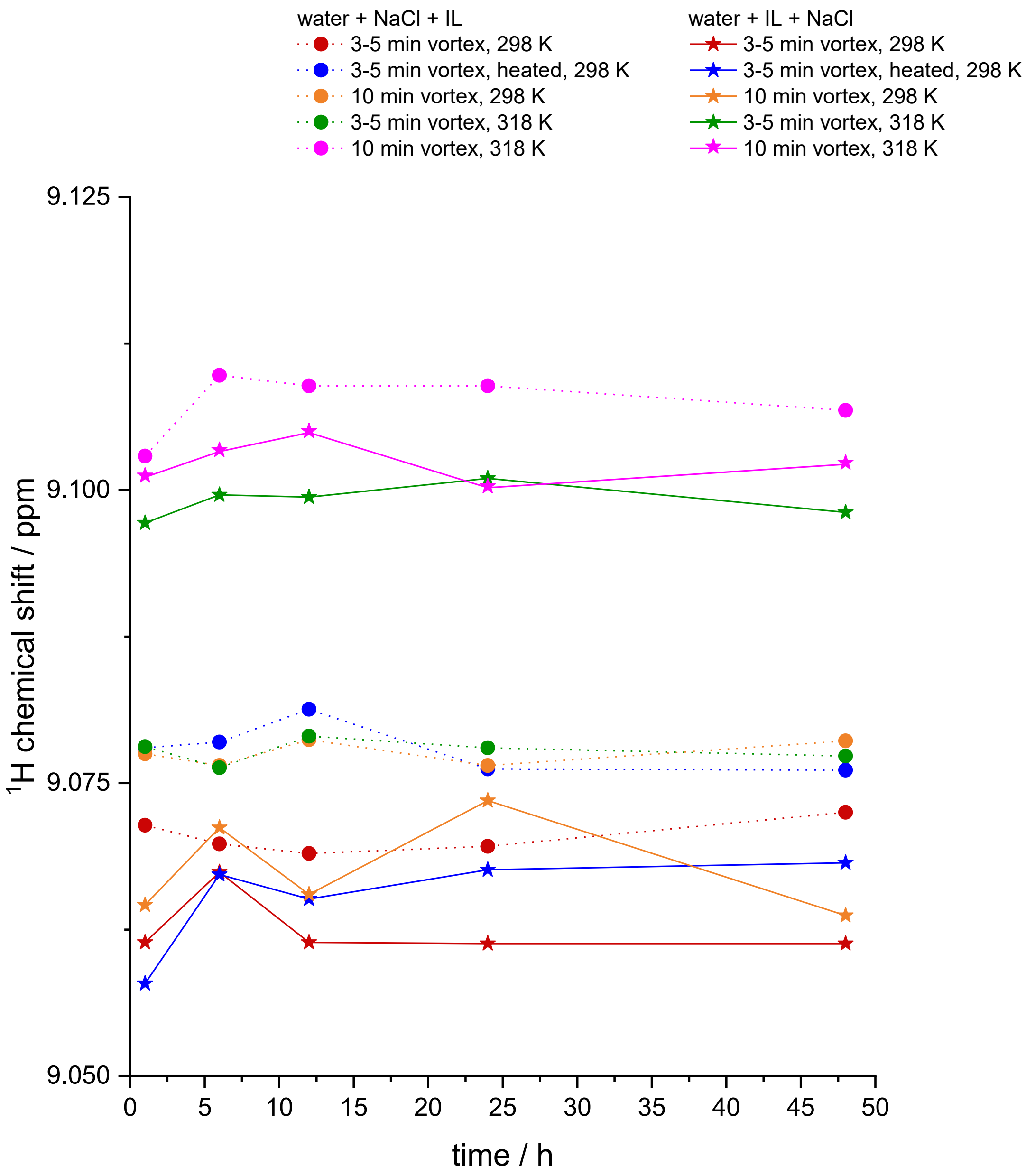

Figure S7. Comparison of ^{1}H chemical shifts (CS) of the aromatic proton H-2, measured in the EMIM-DCA – water – NaCl ternary solutions (X_w = 0.22, 0.1 M NaCl) for different protocols applied during the sample preparation: varying mixing time (3 - 5 min or 10 min), heating of the solution to 318 K, applied before (and not during) the measurement ("heated"), and temperature (measured at 298 K or 318 K). Two mixing orders of the compounds are used: water + NaCl + IL (dot) and water + IL + NaCl (star). The CS are presented as the function of time, representing the age of the sample, selected between 1 h and 48 h.

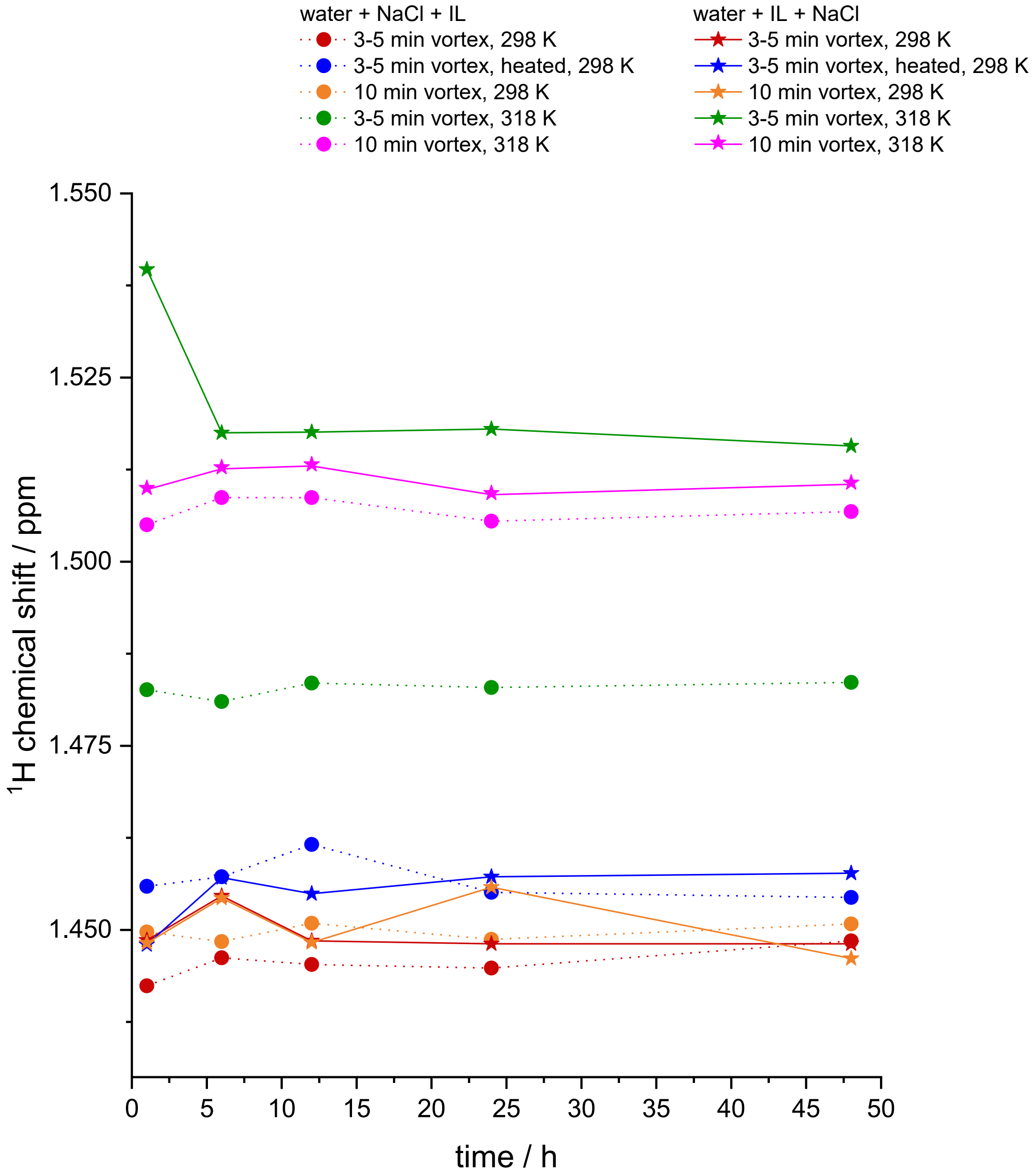

Figure S8. Comparison of ^{1}H chemical shifts (CS) of the aliphatic proton H-8, measured in the EMIM-DCA – water – NaCl ternary solutions (X_w = 0.22, 0.1 M NaCl) for different protocols applied during the sample preparation: varying mixing time (3 - 5 min or 10 min), heating of the solution to 318 K, applied before (and not during) the measurement ("heated"), and temperature (measured at 298 K or 318 K). Two mixing orders of the compounds are used: water + NaCl + IL (dot) and water + IL + NaCl (star). The CS are presented as the function of time, representing the age of the sample, selected between 1 h and 48 h.

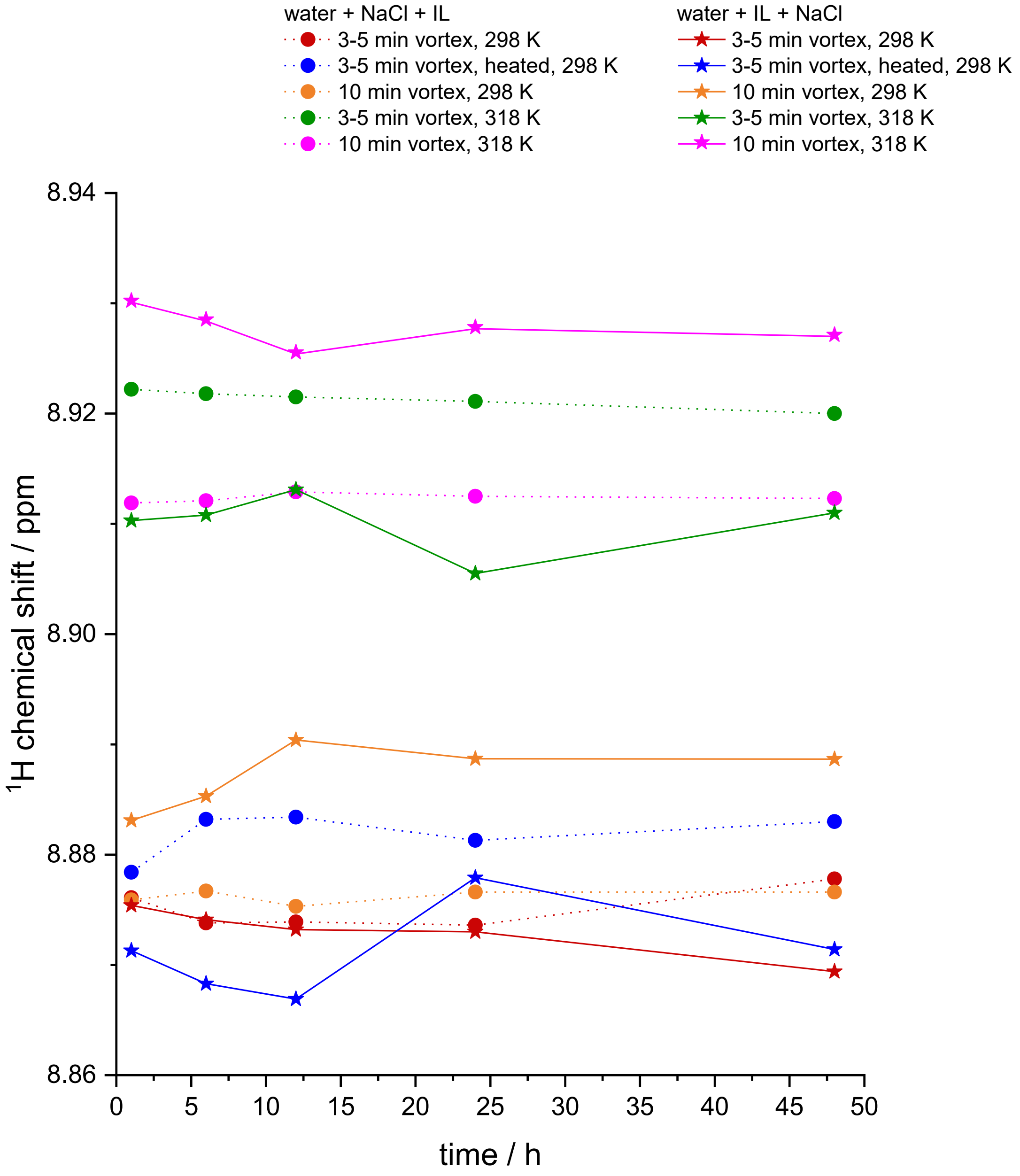

Figure S9. Comparison of ^{1}H chemical shifts (CS) of the aromatic proton H-2, measured in the EMIM-DCA – water – NaCl ternary solutions (X_w = 0.66, 0.1 M NaCl) for different protocols applied during the sample preparation: varying mixing time (3 - 5 min or 10 min), heating of the solution to 318 K, applied before (and not during) the measurement ("heated"), and temperature (measured at 298 K or 318 K). Two mixing orders of the compounds are used: water + NaCl + IL (dot) and water + IL + NaCl (star). The CS are presented as the function of time, representing the age of the sample, selected between 1 h and 48 h.

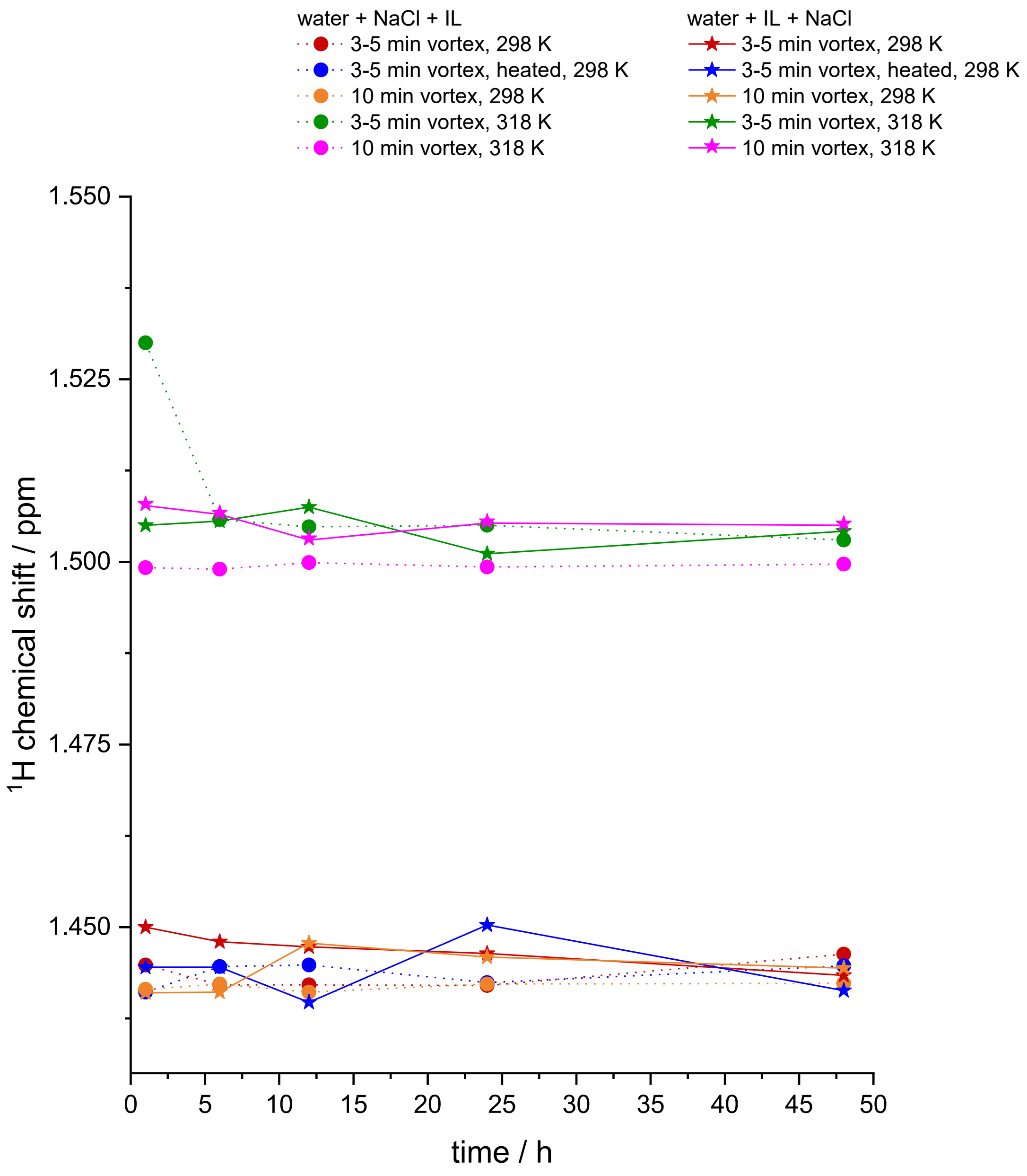

Figure S10. Comparison of ^{1}H chemical shifts (CS) of the aliphatic proton H-8, measured in the EMIM-DCA – water – NaCl ternary solutions (X_w = 0.66, 0.1 M NaCl) for different protocols applied during the sample preparation: varying mixing time (3 - 5 min or 10 min), heating of the solution to 318 K, applied before (and not during) the measurement ("heated"), and temperature (measured at 298 K or 318 K). Two mixing orders of the compounds are used: water + NaCl + IL (dot) and water + IL + NaCl (star). The CS are presented as the function of time, representing the age of the sample, selected between 1 h and 48 h.

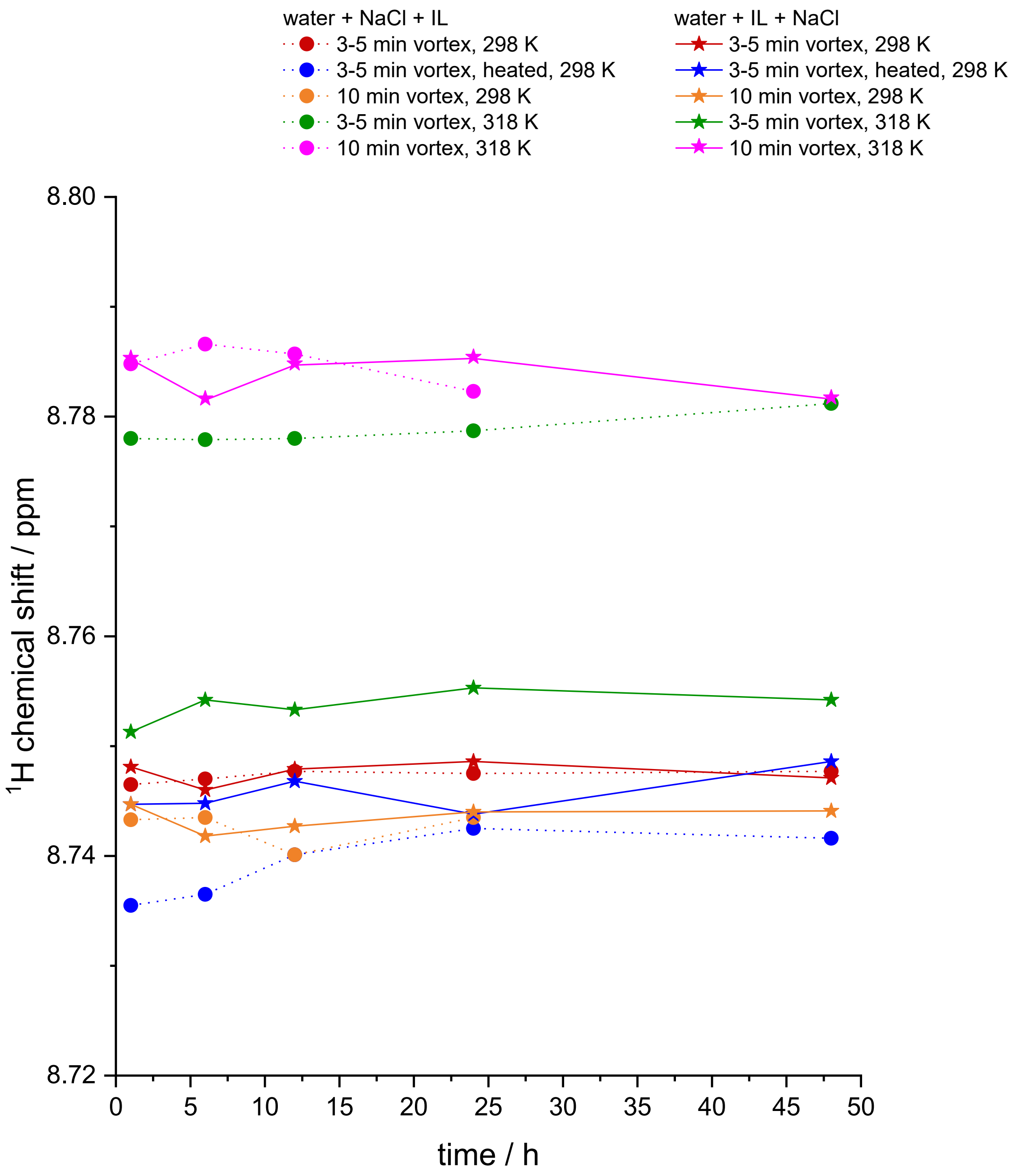

Figure S11. Comparison of ^{1}H chemical shifts (CS) of the aromatic proton H-2, measured in the EMIM-DCA – water – NaCl ternary solutions (X_w = 0.86, 0.1 M NaCl) for different protocols applied during the sample preparation: varying mixing time (3 - 5 min or 10 min), heating of the solution to 318 K, applied before (and not during) the measurement ("heated"), and temperature (measured at 298 K or 318 K). Two mixing orders of the compounds are used: water + NaCl + IL (dot) and water + IL + NaCl (star). The CS are presented as the function of time, representing the age of the sample, selected between 1 h and 48 h.

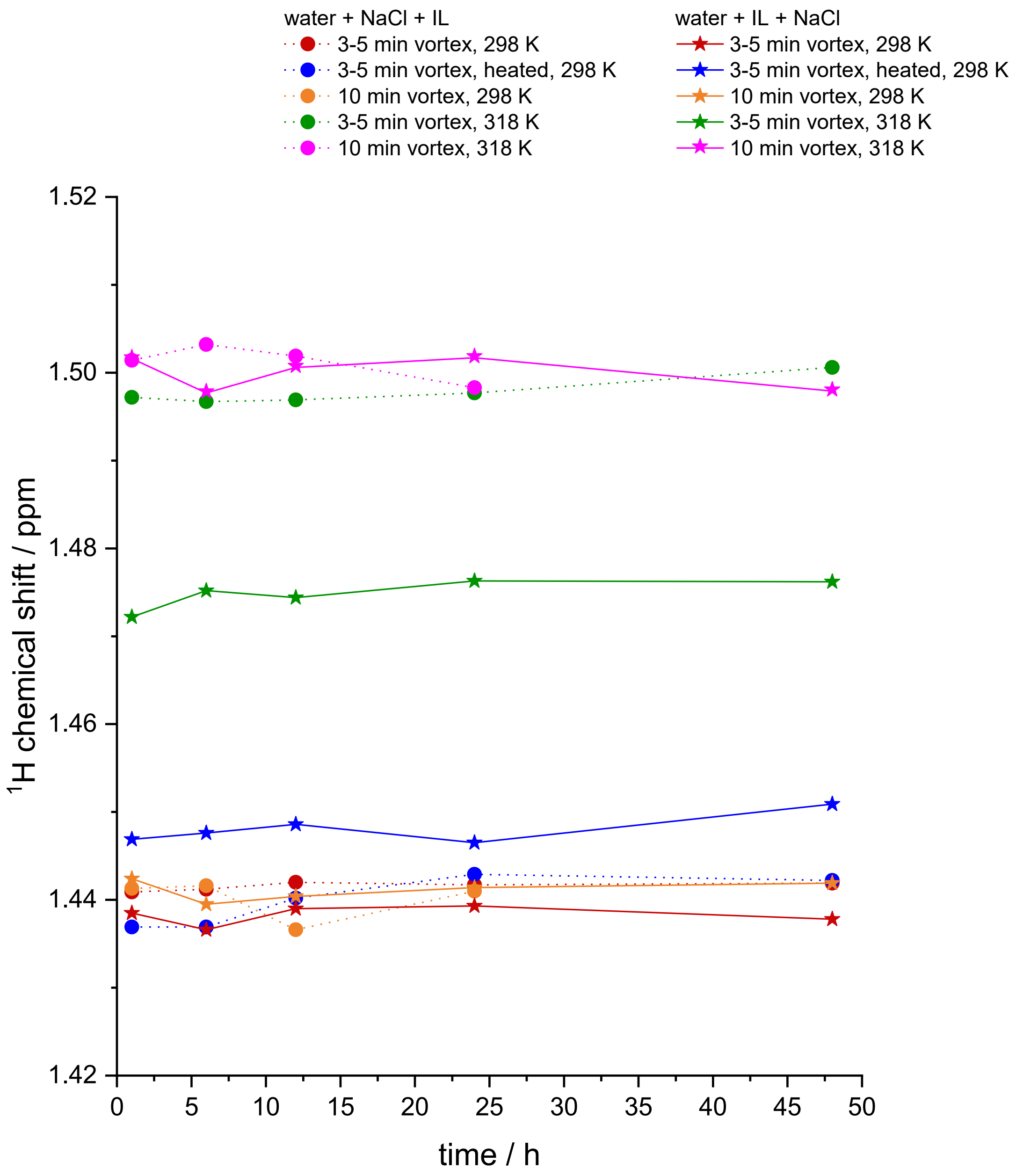

Figure S12. Comparison of ^{1}H chemical shifts (CS) of the aliphatic proton H-8, measured in the EMIM-DCA – water – NaCl ternary solutions (X_w = 0.86, 0.1 M NaCl) for different protocols applied during the sample preparation: varying mixing time (3 - 5 min or 10 min), heating of the solution to 318 K, applied before (and not during) the measurement ("heated"), and temperature (measured at 298 K or 318 K). Two mixing orders of the compounds are used: water + NaCl + IL (dot) and water + IL + NaCl (star). The CS are presented as the function of time, representing the age of the sample, selected between 1 h and 48 h.

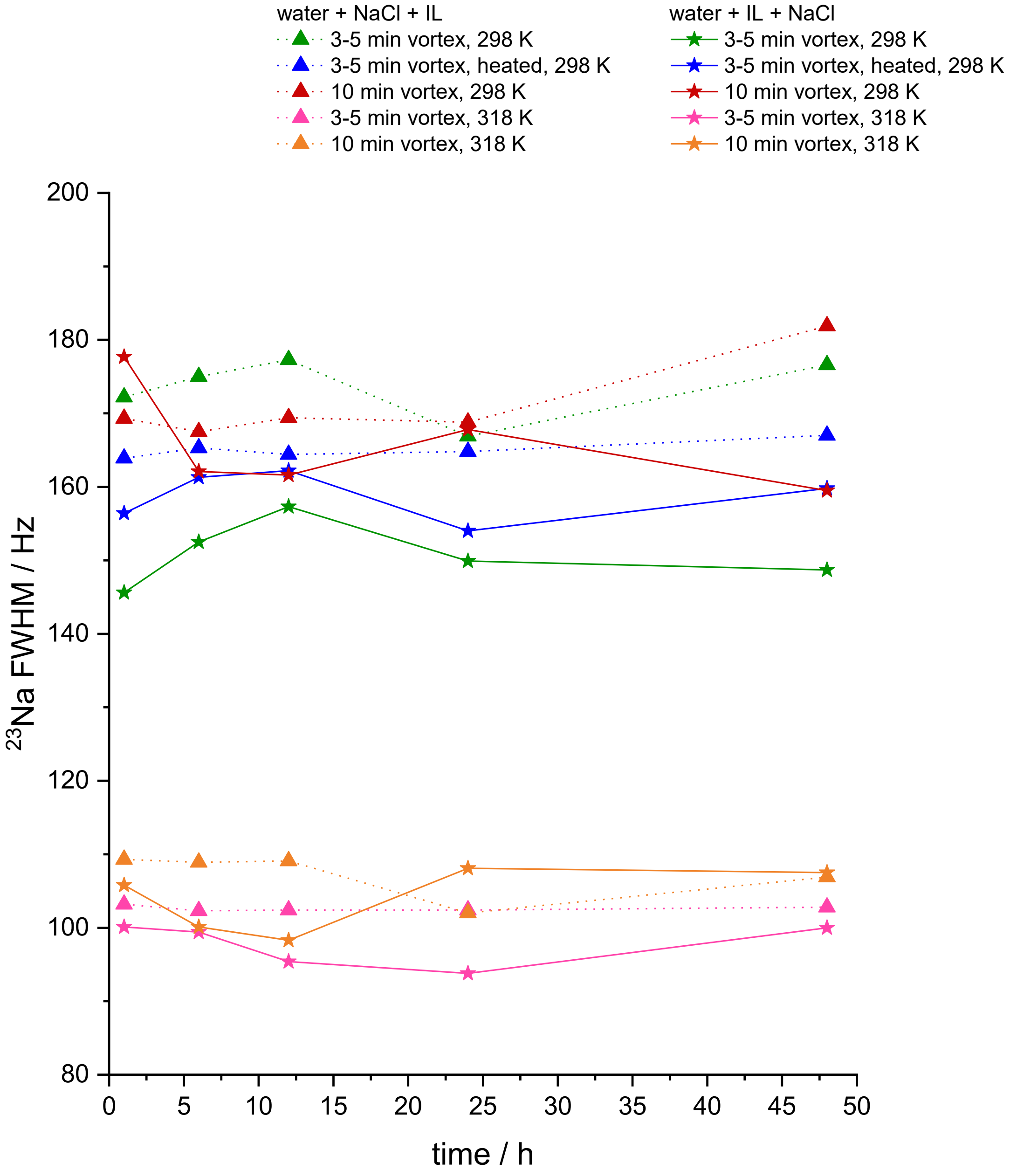

Figure S13. Comparison of ^{23}Na full width at half maximum (FWHM) of peak (**1**), measured in the EMIM-DCA – water – NaCl ternary solutions (X_w = 0.22, 0.1 M NaCl) for different protocols applied during the sample preparation: varying mixing time (3 - 5 min or 10 min), heating of the solution to 318 K, applied before (and not during) the measurement ("heated"), and temperature (measured at 298 K or 318 K). Two mixing orders of the compounds are used: water + NaCl + IL (triangle) and water + IL + NaCl (star). The CS are presented as the function of time, rep-resenting the age of the sample, selected between 1 h and 48 h.

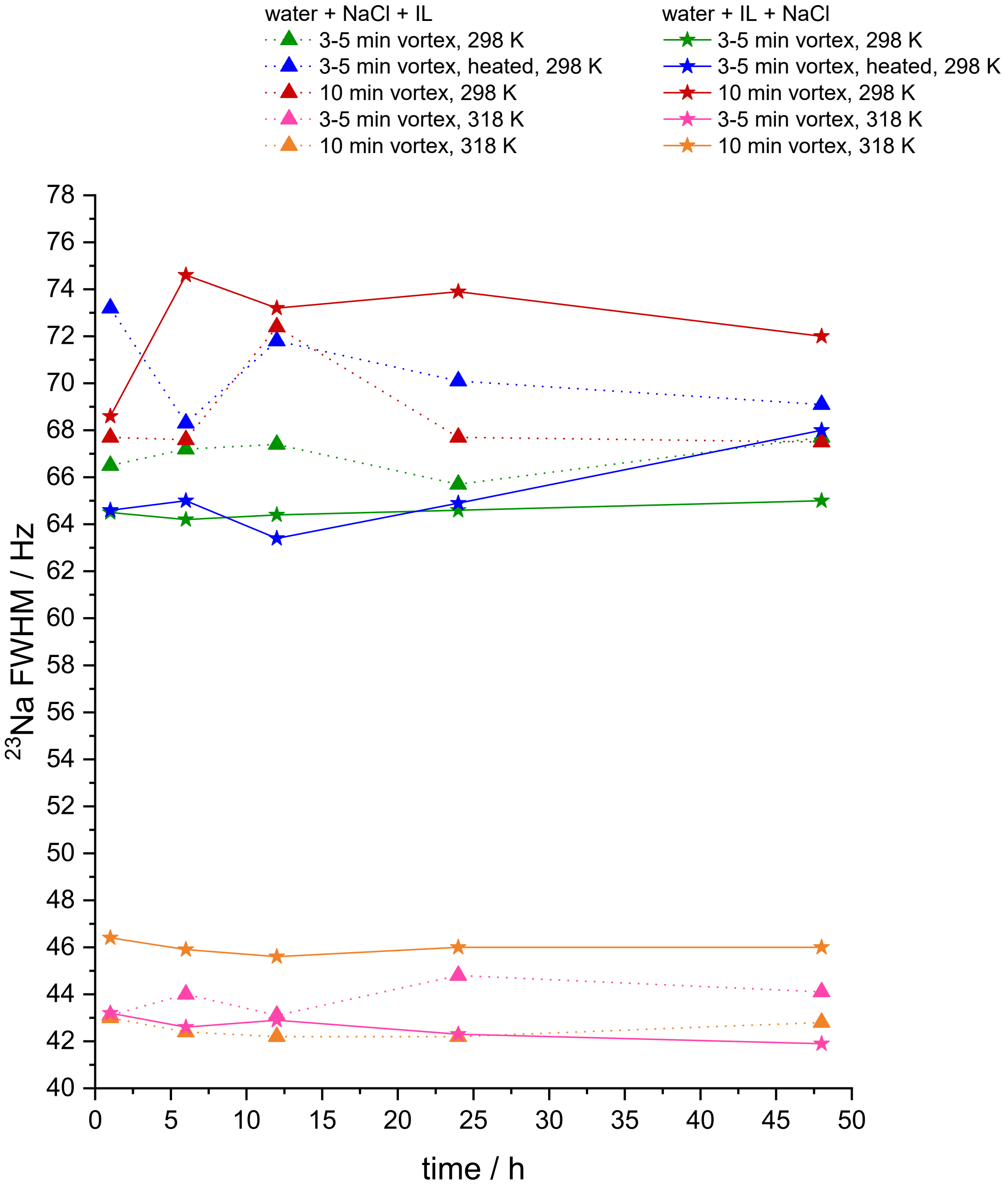

Figure S14. Comparison of ^{23}Na full width at half maximum (FWHM) of peak (**1**), measured in the EMIM-DCA – water – NaCl ternary solutions (X_w = 0.66, 0.1 M NaCl) for different protocols applied during the sample preparation: varying mixing time (3 - 5 min or 10 min), heating of the solution to 318 K, applied before (and not during) the measurement ("heated"), and temperature (measured at 298 K or 318 K). Two mixing orders of the compounds are used: water + NaCl + IL (triangle) and water + IL + NaCl (star). The CS are presented as the function of time, rep-resenting the age of the sample, selected between 1 h and 48 h.

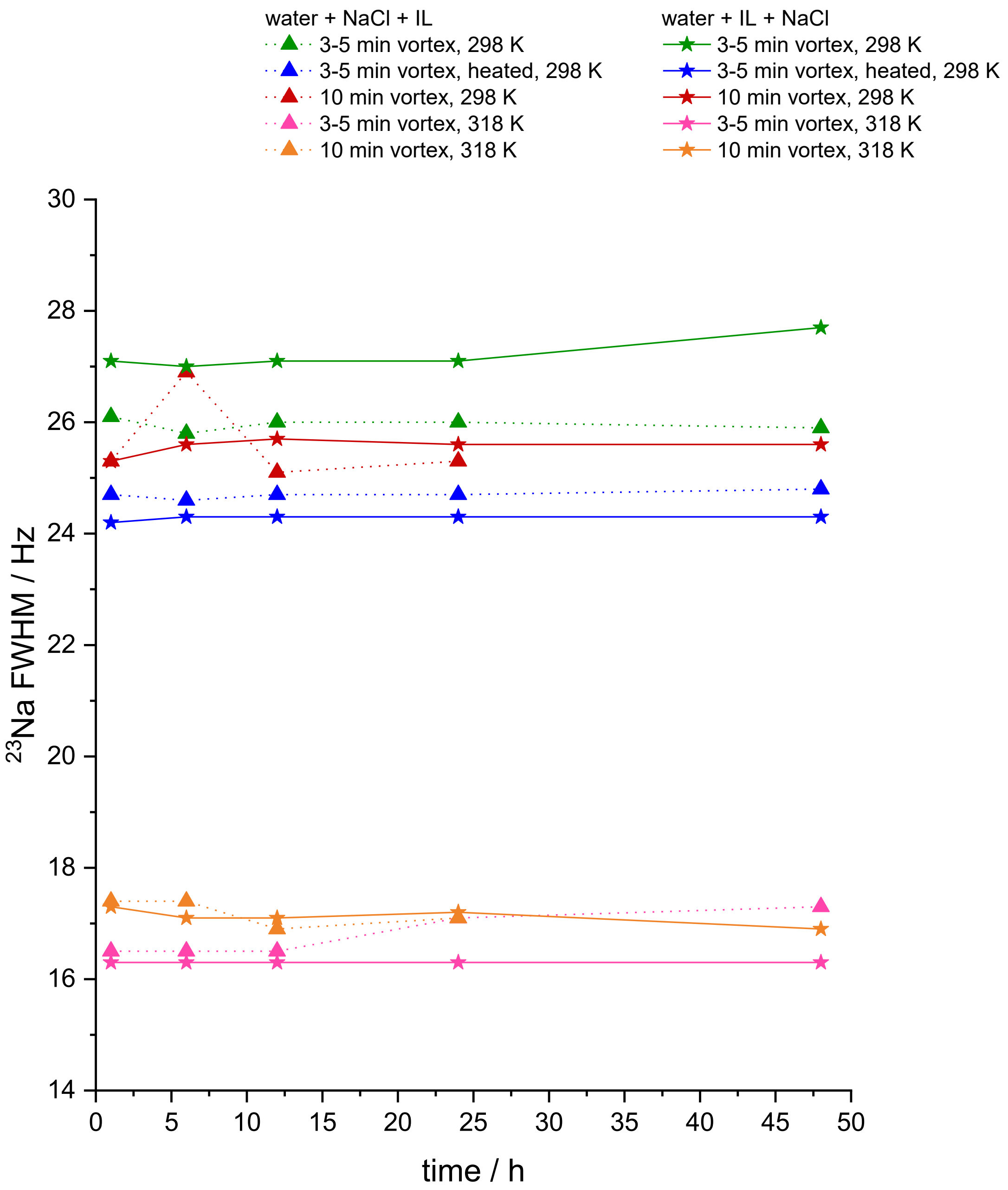

Figure S15. Comparison of ^{23}Na full width at half maximum (FWHM) of peak (**1**), measured in the EMIM-DCA – water – NaCl ternary solutions (X_w = 0.66, 0.1 M NaCl) for different protocols applied during the sample preparation: varying mixing time (3 - 5 min or 10 min), heating of the solution to 318 K, applied before (and not during) the measurement ("heated"), and temperature (measured at 298 K or 318 K). Two mixing orders of the compounds are used: water + NaCl + IL (triangle) and water + IL + NaCl (star). The CS are presented as the function of time, rep-resenting the age of the sample, selected between 1 h and 48 h.

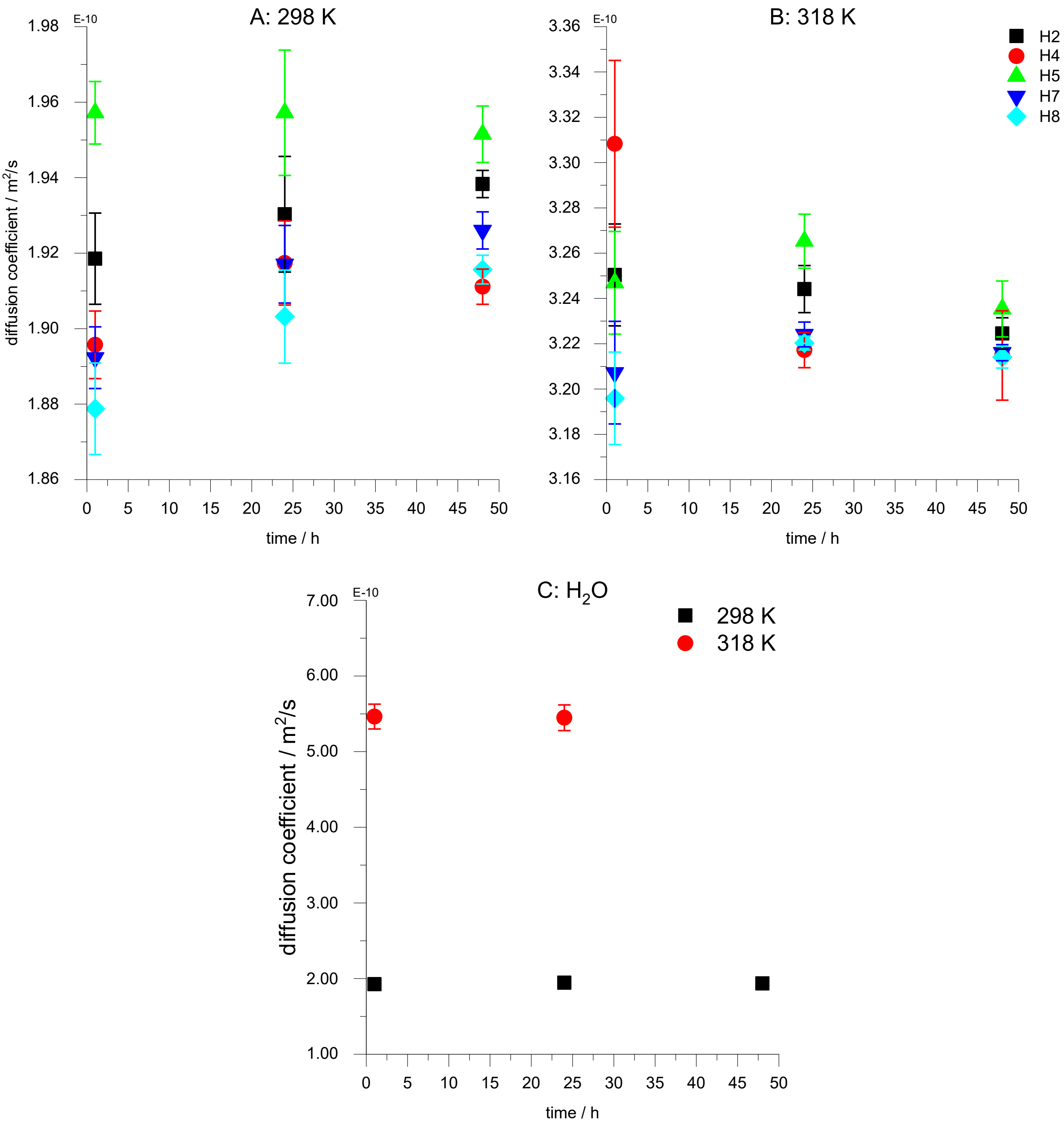

Figure S16. Diffusion coefficients of EMIM$^+$ and water in ternary solution (X_w = 0.45, 0.1 M NaCl) with the mixing order: water + NaCl + IL, determined for EMIM$^+$ at 298 K (**A**), EMIM$^+$ at 318 K (**B**) and water at 298 K and 318 K (**C**) as a function of time.

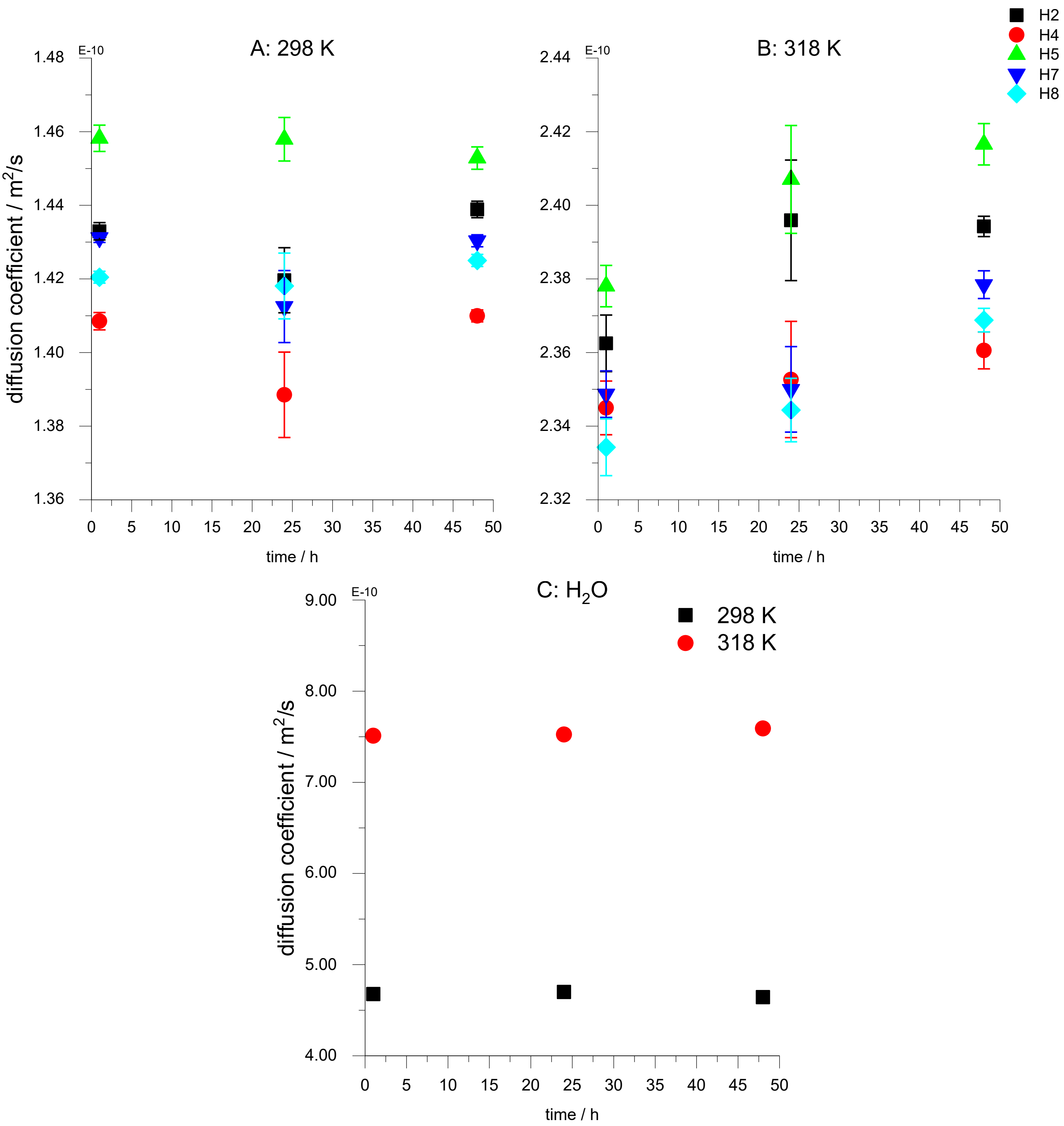

Figure S17. Diffusion coefficients of $EMIM^+$ and water in ternary solution (X_w = 0.45, 0.1 M NaCl) with the mixing order: water + IL + NaCl, determined for $EMIM^+$ at 298 K (**A**), $EMIM^+$ at 318 K (**B**) and water at 298 K and 318 K (**C**) as a function of time.

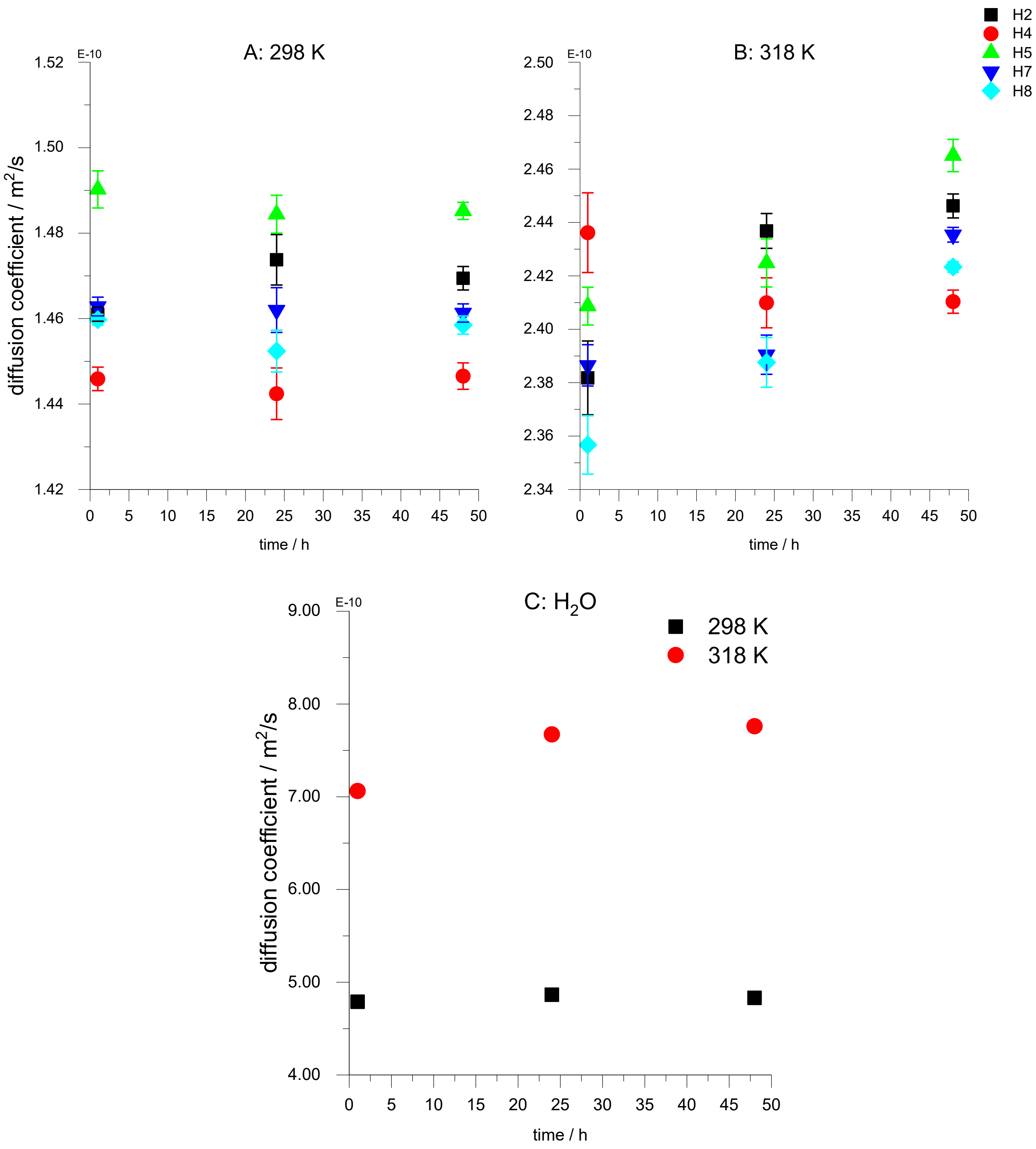

Figure S18. Diffusion coefficients of $EMIM^+$ and water in ternary solution (X_w = 0.45, 0.1 mM NaCl) with the mixing order: water + NaCl + IL, determined for $EMIM^+$ at 298 K (**A**), $EMIM^+$ at 318 K (**B**) and water at 298 K and 318 K (**C**) as a function of time.

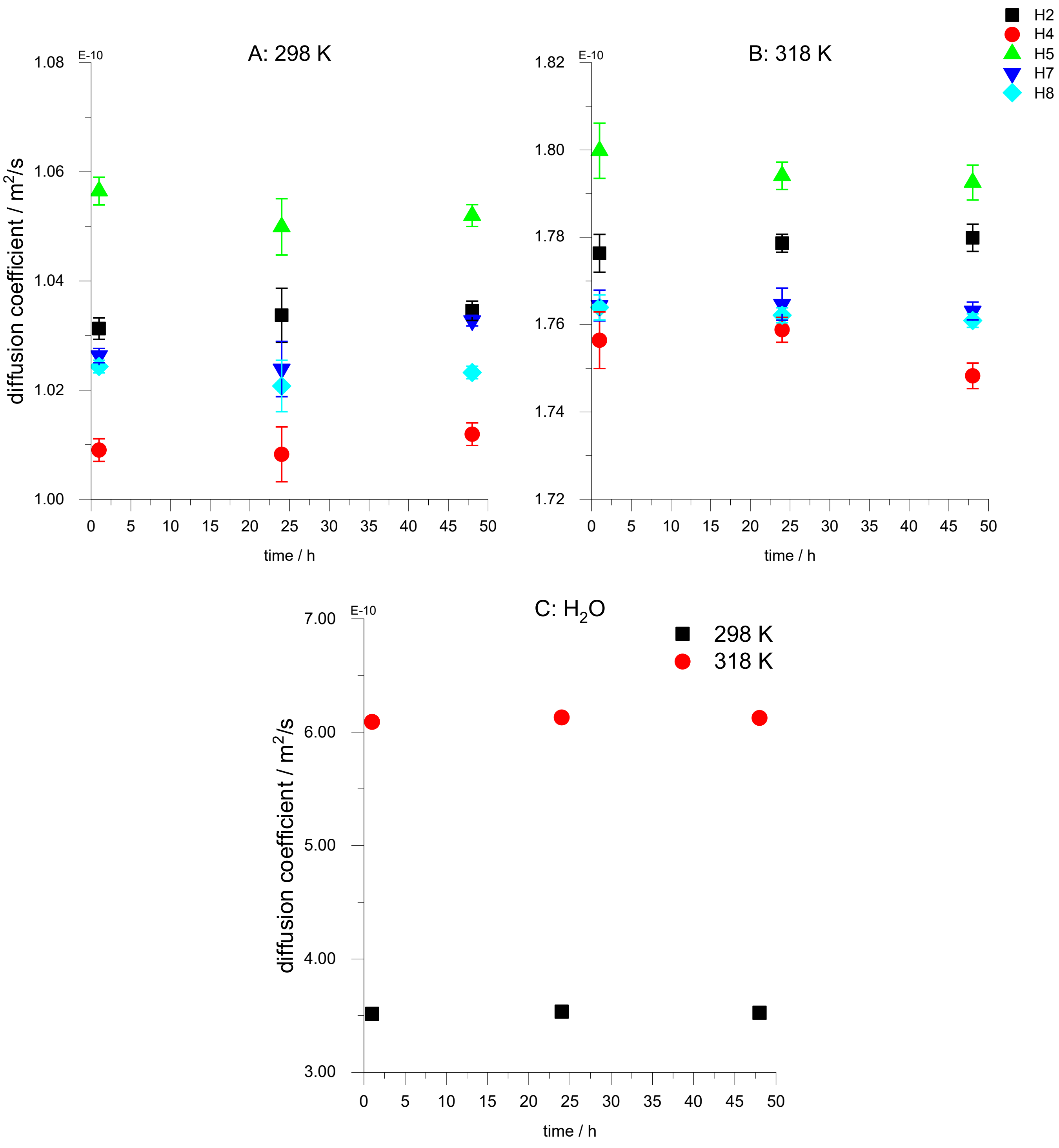

Figure S19. Diffusion coefficients of $EMIM^+$ and water in ternary solution ($X_w = 0.24$, 1:0.022 water:NaCl molecular ratio) with the mixing order: water + NaCl + IL, determined for $EMIM^+$ at 298 K (**A**), $EMIM^+$ at 318 K (**B**) and water at 298 K and 318 K (**C**) as a function of time.

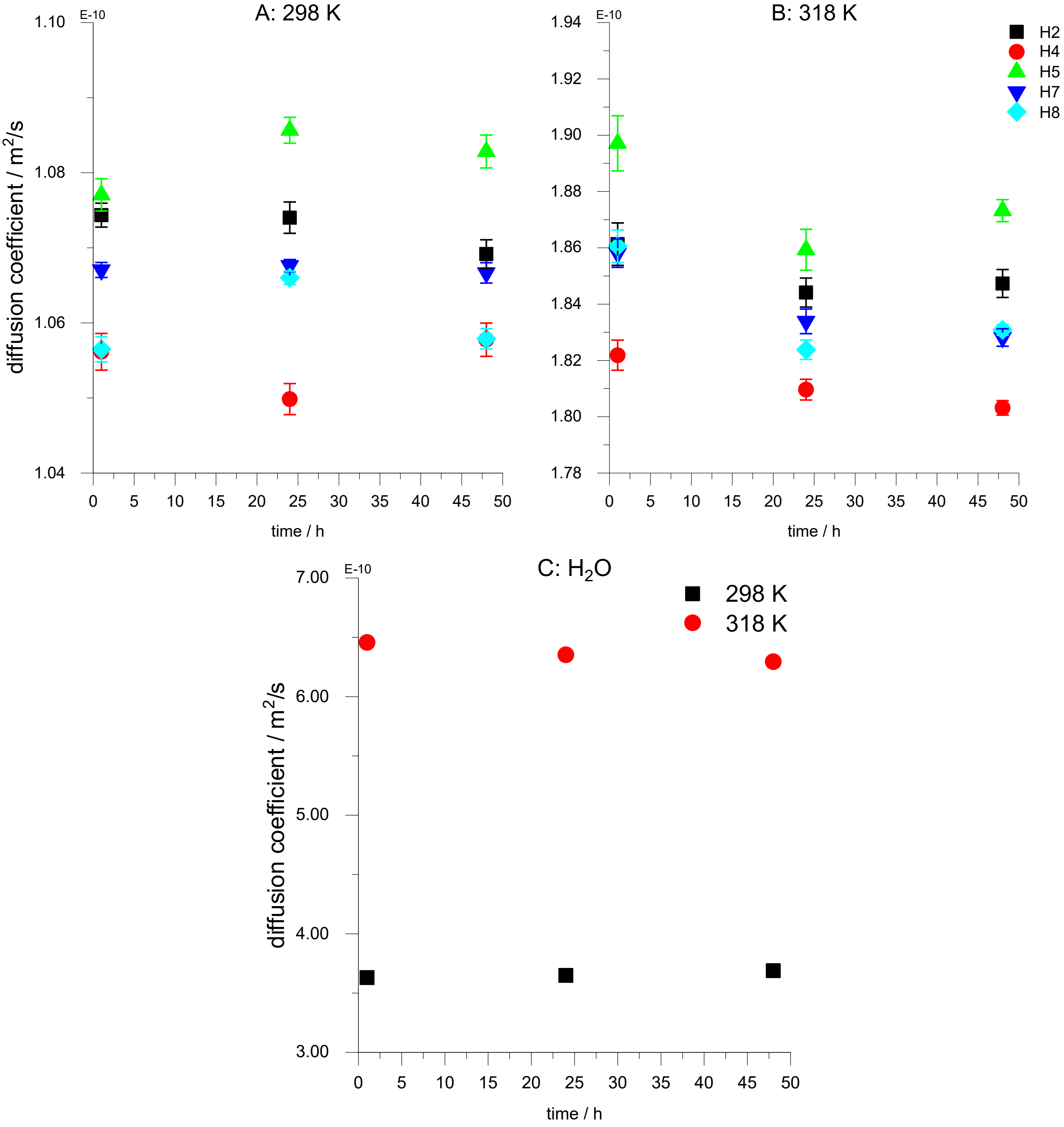

Figure S20. Diffusion coefficients of $EMIM^+$ and water in ternary solution ($X_w = 0.24$, 1:0.022 water:NaCl molecular ratio) with the mixing order: water + IL + NaCl, determined for $EMIM^+$ at 298 K (**A**), $EMIM^+$ at 318 K (**B**) and water at 298 K and 318 K (**C**) as a function of time.

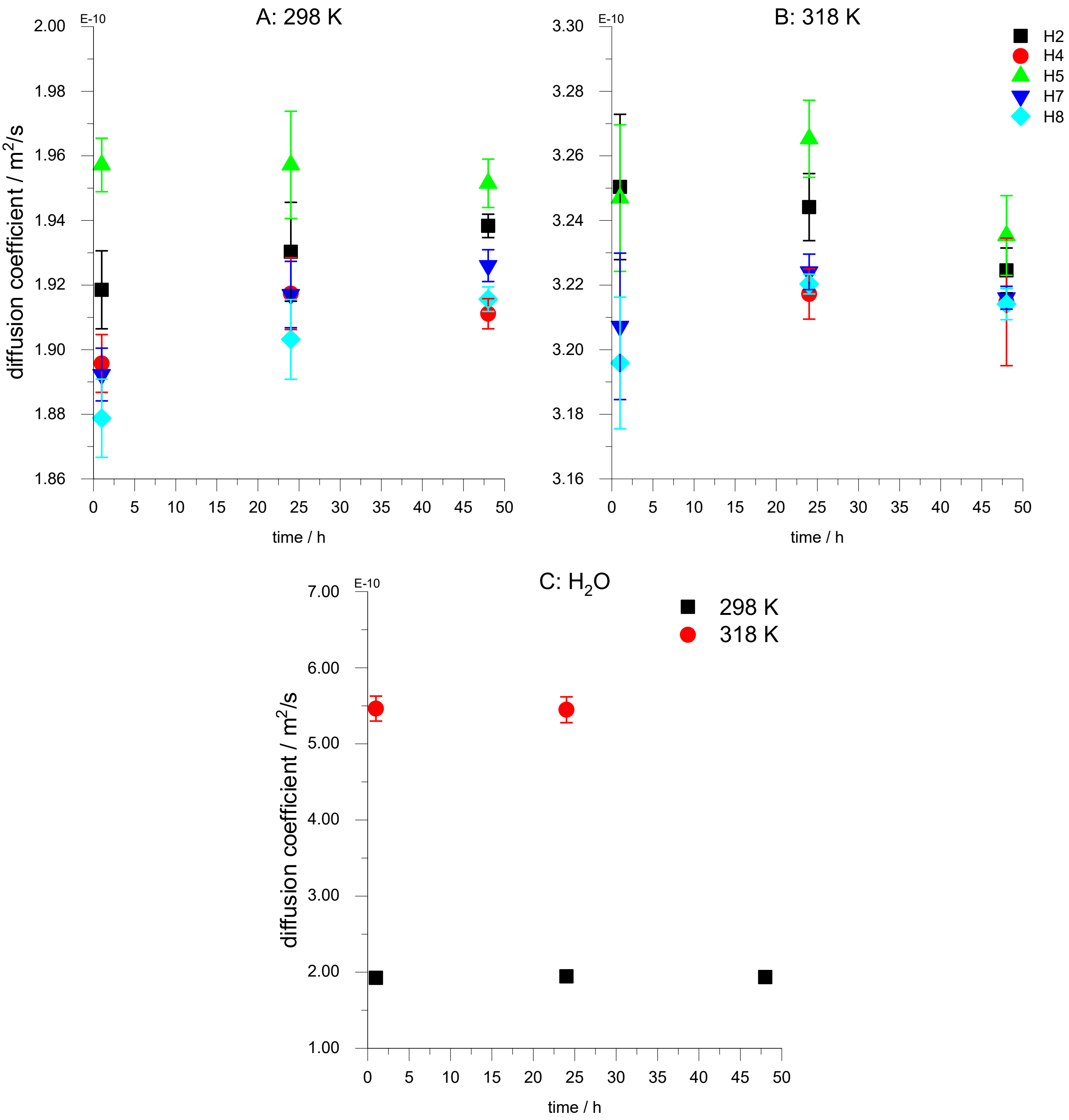

Figure S21. Diffusion coefficients of $EMIM^+$ and water in ternary solution ($X_w = 0.67$, 1:0.022 water:NaCl molecular ratio) with the mixing order: water + NaCl + IL, determined for $EMIM^+$ at 298 K (**A**), $EMIM^+$ at 318 K (**B**) and water at 298 K and 318 K (**C**) as a function of time.

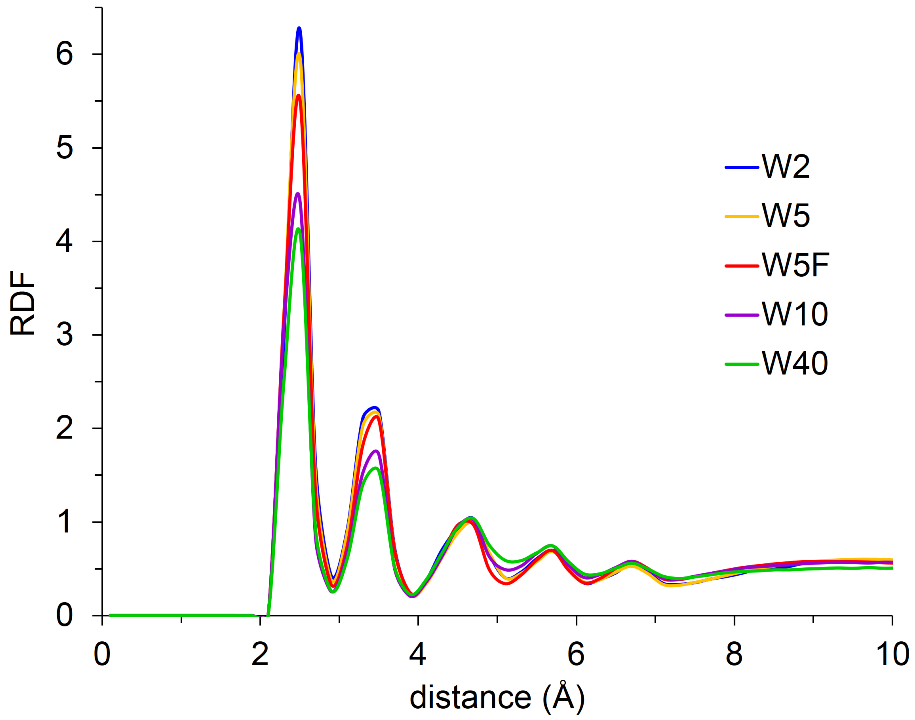

Figure S22. Radial distribution function of the DCA anion with respect to the sodium ion for all trajectories.

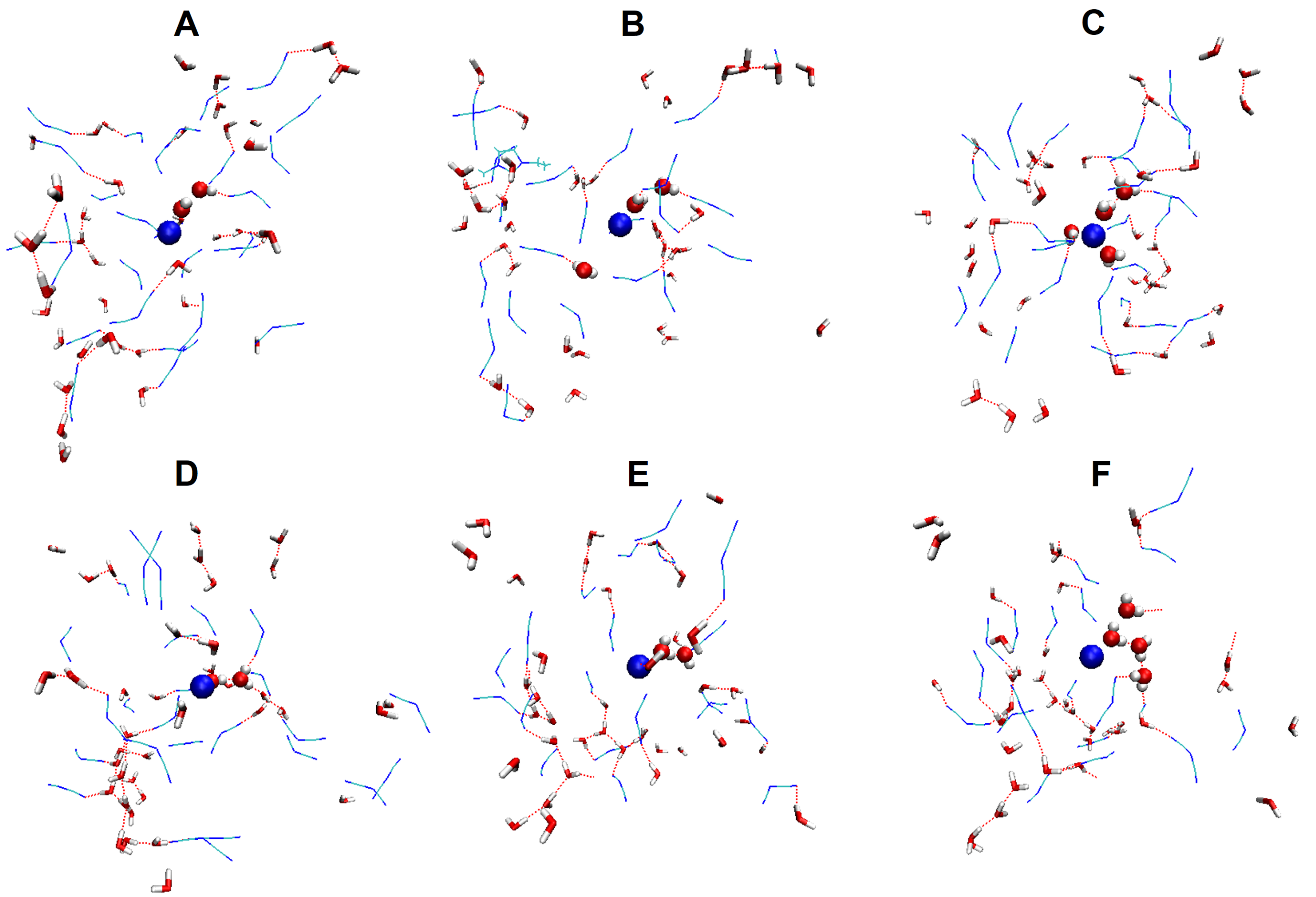

Figure S23. Network of hydrogen bonds between water molecules and ILs at different simulation time. Water molecules in the first hydration shell are shown by a sphere model. Sodium cation is shown by the blue sphere model while the DCA and EMIM ions are represented by the line model. The color code is sodium: blue, oxygen: red, hydro-gen: white, carbon: cyan, and nitrogen: blue.
